# State of the Art of CAR-NK Cell Therapy in Multiple Myeloma: A Comprehensive Review of Cell Sources and Target Antigens

**DOI:** 10.3390/ijms262211224

**Published:** 2025-11-20

**Authors:** Asya Bastrich, Kamilla Vinogradova, Diana Mokrousova, Anna Efremova, Oleg Makhnach, Dmitry Goldshtein

**Affiliations:** Research Centre for Medical Genetics, 1 Moskvorechye St., Moscow 115522, Russia; asjaha@gmail.com (A.B.); v.kamilla.v@yandex.com (K.V.); diana-mok2000@yandex.ru (D.M.); buben6@yandex.ru (O.M.); dvgoldshtein@gmail.com (D.G.)

**Keywords:** CAR-NK cells, multiple myeloma, immunotherapy, BCMA, GPRC5D, NKG2D, CD38, SLAMF7, CD138, iPSC-derived NK cells, allogeneic cell therapy

## Abstract

Multiple myeloma (MM) is a clonal malignancy of plasma cells that remains largely incurable despite major advances in proteasome inhibitors, immunomodulatory drugs, and monoclonal antibodies. Chimeric antigen receptor (CAR)-engineered immune cells have transformed the therapeutic landscape, but CAR-T cell therapy faces challenges such as severe cytokine release syndrome (CRS), neurotoxicity, limited persistence, and logistical complexity. In recent years, natural killer (NK) cells have emerged as a promising platform for next-generation cellular immunotherapy, offering innate antitumor activity, a reduced risk of graft-versus-host disease (GvHD), and the feasibility of “off-the-shelf” allogeneic production. This review summarizes current advances in CAR-NK cell therapy for MM, focusing on two major aspects: the diversity of cell sources—including NK-92, peripheral (PB) and cord blood (CB), and induced pluripotent stem cell (iPSC)-derived NK cells—and the expanding repertoire of target antigens such as BCMA (B-cell maturation antigen), NKG2D, CD38, CD70, SLAMF7, CD138, and GPRC5D. We highlight preclinical and early clinical studies demonstrating potent cytotoxicity, favorable safety profiles, and innovative multi-targeting strategies designed to overcome antigen escape and enhance persistence. Emerging clinical data suggest that CAR-NK cell therapy may combine the specificity of CAR recognition with the inherent safety and versatility of NK biology, offering a potential paradigm shift in the treatment of relapsed or refractory MM. Further clinical validation will determine whether CAR-NK cell therapy can achieve durable remission and complement or surpass current CAR-T modalities.

## 1. Introduction

MM is a hematologic malignancy of clonal plasma cells [1,2,3] that remains largely incurable despite major therapeutic advances in proteasome inhibitors, immunomodulatory drugs, and monoclonal antibodies [4]. Although these agents have extended survival, most patients eventually relapse, highlighting the need for novel and durable immunotherapeutic strategies [5].

CAR-T cell therapies targeting BCMA have achieved remarkable responses in relapsed/refractory MM [6,7]. However, their use is constrained by severe CRS, immune effector cell–associated neurotoxicity syndrome (ICANS), prolonged cytopenia, and manufacturing constraints [8,9]. Furthermore, the emergence of antigen loss variants and the immunosuppressive tumor microenvironment often result in relapse, underscoring the demand for safer, “off-the-shelf”, and more persistent cellular platforms.

NK cells offer a compelling alternative. As innate cytotoxic lymphocytes capable of killing malignant cells independently of antigen presentation, NK cells can be engineered with CAR constructs to combine intrinsic tumor surveillance with antigen-specific targeting. Compared with CAR-T cells, CAR-NK products exhibit a reduced risk of CRS and GvHD, and can be generated from allogeneic sources, enabling scalable “off-the-shelf” therapies [10,11,12]. Recent preclinical and early clinical data demonstrate that CAR-NK cells targeting antigens such as BCMA, SLAMF7, CD38, and GPRC5D effectively eliminate MM cells while maintaining a favorable safety profile. These findings position CAR-NK therapy as a promising next-generation approach in the evolving immunotherapy landscape of MM.

In this review, we summarize the current state of CAR-NK cell therapy in MM, emphasizing diverse cell sources and target antigens, and discuss the translational opportunities and challenges shaping their future clinical application.

## 2. Biological Distinctions Between NK Cells and T Cells

Although both T cells and NK cells derive from common lymphoid progenitors [13], they belong to distinct arms of the immune system and exhibit fundamentally different modes of activation and persistence. T cells are hallmarks of the adaptive immune system, requiring antigen presentation via major histocompatibility complex (MHC) molecules for activation [14]. Upon stimulation, T cells undergo clonal expansion and can acquire long-lived central and effector memory phenotypes, enabling robust proliferation and durable immune surveillance in vivo [15]. These stem-like memory properties underlie the potent and sustained responses observed with CAR-T therapies [16].

In contrast, NK cells are innate cytotoxic lymphocytes that do not rely on antigen-specific priming [17]. They recognize and eliminate transformed or stressed cells through a dynamic balance of activating and inhibitory receptor signals [18], allowing rapid, antigen-independent cytotoxicity [19]. However, NK cells generally display limited proliferative capacity and a relatively short lifespan after infusion, which may restrict the persistence and long-term efficacy of CAR-NK products [20].

These intrinsic biological differences explain why CAR-NK therapies are typically associated with improved safety profiles—including reduced CRS and GvHD—yet may exhibit lower in vivo expansion and durability compared with CAR-T cells. Understanding these fundamental distinctions provides essential context for optimizing CAR design, cell engineering, and manufacturing strategies tailored to the NK platform.

## 3. Chimeric Antigen Receptor Technology as One of the Strategies to Improve NK Cell Anti-Multiple Myeloma Response

**Figure 1:**
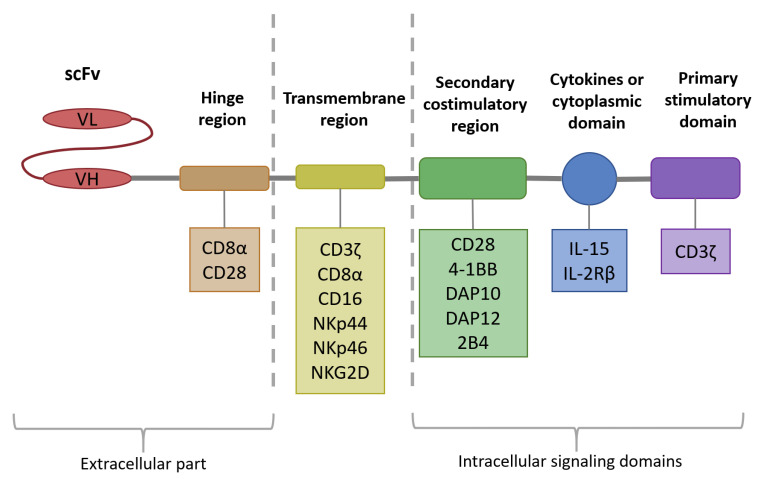
CAR-NK construct design.

CARs are synthetic fusion proteins composed of several functional modules, designed to specifically recognize antigens expressed on the surface of target cells (Figure 1). They include an extracellular antigen-recognition region that binds tumor-associated antigens (TAA), typically represented by a single-chain variable fragment (scFv) of an immunoglobulin; a hinge region that provides structural flexibility; a transmembrane domain (TMD) anchoring the receptor to the cell membrane; and intracellular signaling domains that initiate immune cell activation and subsequent tumor cell elimination [21]. Thus, CAR technology enables the ex vivo reprogramming of immune effector cells, combining, on the one hand, the advantages of a monoclonal antibody with high affinity and MHC class I–independent recognition, and, on the other hand, the cytotoxic potential of lymphocytes capable of proliferation and sustained antitumor activity. Applied to NK cells, this strategy provides an alternative form of adoptive immunotherapy, in which CAR-NK cells retain their intrinsic ability to recognize and eliminate malignant cells while gaining an additional layer of antigen specificity through CAR-mediated targeting.

### 3.1. Intracellular Signaling Domain

To date, five generations of CAR constructs have been described, differing primarily in the design of the intracellular signaling domain [22]. Within the intracellular region of CARs, the signaling architecture consists of a primary stimulatory domain, responsible for initiating effector cell activation, and may also incorporate secondary costimulatory domains that enhance and sustain cellular responses. As the primary stimulatory domain, CAR-NK engineered to target MM antigens most commonly utilize CD3ζ, which contains immunoreceptor tyrosine-based activation motifs.

It should be noted that the concept of CAR “generations” is largely conceptual. While all of these designs have been described, their practical application in CAR-NK cell development remains uneven, with much of the current knowledge originating from CAR-T research. The extent to which such findings can be directly extrapolated to CAR-NK platforms has yet to be conclusively demonstrated, and examples specifically addressing MM models are especially limited. Accordingly, in this review we have aimed to focus on evidence derived directly from CAR-NK studies, highlighting cases relevant to MM where available.

First-generation CARs consist of a hybrid receptor comprising an extracellular domain for tumor antigen recognition, a TMD, and an intracellular signaling domain capable of triggering NK cell activation. Due to the absence of costimulatory domains, first-generation CARs demonstrated inefficient for clinical application. An exception is represented by NKG2D-based CARs, which, through interaction with their natural ligands, provide intrinsic costimulatory signaling that operates independently [23].

Second- [24] and third-generation CARs [25] incorporate one or two additional intracellular costimulatory domains, respectively, such as CD28 or 4-1BB (CD137), enhancing NK cell effector functions such as cytokine production and target MM cell lysis. Furthermore, the intracellular signaling modules of CARs can be engineered to incorporate more specific costimulatory regions such as DNAX-activating protein 10 (DAP10), DNAX-activating protein 12 (DAP12) and 2B4. DAP12 serves as the essential adaptor for several activating NK receptors, including NKG2C, NKp44, and certain activating KIRs [26], whereas DAP10 is specifically required to mediate the costimulatory signaling of NKG2D [27]. These adaptors naturally associate with activating receptors on NK cells and initiate downstream signaling cascades tailored to NK effector functions, including cytotoxicity and cytokine production. Incorporating DAP10 or DAP12 into CAR constructs may therefore enhance NK cell activation more physiologically than CD3ζ alone, potentially improving anti-myeloma efficacy and persistence while maintaining NK-specific functional programs [28]. For instance, in the context of solid tumor models, CAR constructs incorporating NK-specific costimulatory domains, such as 2B4, DAP10, or DAP12, are characterized by increased cytotoxic activity and elevated IFN-γ secretion, compared with conventional CAR architectures originally tailored for T cells [29]. Besides that, evidence from models of hematologic malignancies has demonstrated that incorporation of the NK-specific costimulatory domain 2B4 into CAR constructs markedly enhances NK cell performance. Specifically, 2B4-based CAR-NK cells exhibited accelerated proliferation, improved antigen-specific cytotoxicity, and superior antitumor efficacy both in vitro [30] and in vivo [31].

Fourth-generation CARs, analogous to second-generation constructs, are further engineered to include transgenes encoding cytokines, thereby enhancing NK cell proliferation, persistence, and cytotoxicity [32]. This strategy has already been validated in preclinical models of MM. For instance, BCMA-CD28-IL15 CAR-NK cells demonstrated more sustained cytotoxic activity and superior control of tumor growth in vivo compared with CAR-NK cells lacking IL-15 [24]. Moreover, the concept of “armored” CAR-NK cells has been clinically demonstrated in the case of CB-derived CD19-CAR-NK cells engineered to express IL-15, where infusions proved safe and resulted in both clinical responses and prolonged cellular persistence in patients with lymphoproliferative disorders [33]. Taken together, these findings indicate that fourth-generation CAR-NK cells represent not only a theoretical advancement but also a feasible therapeutic platform, with clear potential for application in MM treatment.

Fifth-generation CARs also build upon second-generation scaffolds but integrate a cytoplasmic domain derived from IL-2Rβ with STAT3/5 recruitment motifs, further fine-tuning intracellular signaling and promoting NK-specific functional responses. These design principles remain predominantly at the conceptual stage in the context of MM, underscoring their status as an emerging and still largely unexplored avenue within CAR-NK-cell-based immunotherapy.

### 3.2. Transmembrane Domain

The TMD is a single-pass lipophilic α-helical segment of CAR within the NK cell membrane. It serves as a structural bridge between the extracellular and intracellular regions of the CAR. It contributes to the proper expression, anchoring, and stabilization of the receptor within the cell membrane, while also ensuring effective signal transmission required for CAR-mediated activation. The choice of transmembrane element significantly impacts receptor stability and the magnitude of downstream signaling. Similarly to CAR-T designs, the most widely applied TMDs in CAR-NK constructs are derived from CD3ζ, CD8α, and CD28 [25]. In addition multiple CAR configurations have been tested in NK cells, incorporating a range of transmembrane regions from NK cell receptors such as CD16, NKp44, NKp46, and NKG2D [34]. Evidence suggests that NK-specific domains, for example, those derived from NKG2D, can enhance degranulation, as reflected by increased CD107a expression, and promote cytotoxic activity, with these effects confirmed in both in vitro and in vivo models of CD19^+^ lymphoma [30]. These observations point to a potential functional advantage of NK-adapted transmembrane elements in CAR design. However, the extent to which such domains provide clear benefits over conventional counterparts has not been systematically examined [34], and examples specifically demonstrated in MM models remain very limited.

### 3.3. Hinge Region

The hinge region, also referred to as the spacer, is a short extracellular segment that connects the antigen-recognition domain to the TMD in CAR-NK constructs. Its primary function is to provide sufficient spatial flexibility to the antigen-recognition region, enabling optimal engagement with target antigens such as BCMA, which is frequently employed in CAR-NK designs for MM. Notably, in NK cells, the choice of hinge can significantly affect immune synapse formation, as NK effector functions depend on precise receptor clustering and signaling. For instance, overly long IgG-derived hinges may introduce unintended interactions via Fcγ receptor binding. This is particularly relevant because, unlike T cells, NK cells express FcγRIII (CD16), potentially leading to off-target activation. Therefore, optimizing hinge length and origin is crucial when designing CAR constructs [35]. More rigid hinges, such as those derived from CD8α or CD28, promote effective synapse formation and enhance target engagement in CAR-NK cells. For example, BCMA-CD28-IL15 CAR-NK cells demonstrated superior cytotoxic activity and secreted significantly higher levels of IFN-γ compared with BCMA-hIgG1-IL15 CAR-NK cells [24].

Similarly to CAR-T cells, CAR-NK cells adopt a modular architecture consisting of an antigen-binding domain, a hinge, a TMD, and intracellular signaling domain, with each module offering opportunities for targeted design and optimization. Over the years, CAR architecture has been refined through advances in molecular engineering and immunotherapy, and strategies tailored to the unique signaling machinery of NK cells have emerged as particularly promising. Such approaches aim to enhance cytotoxic activity, cytokine secretion, and in vivo persistence, thereby increasing the therapeutic potential of CAR-NK cells in MM. In this context, the genetic engineering of NK cells with CARs has gained growing attention and has been investigated extensively in vitro and in vivo over the past two decades. These efforts encompass the use of different NK cell sources, varied methods for culture, expansion, and transduction, as well as diverse plasmid constructs and vector systems. As a result, a number of CAR-NK cell candidates for MM are currently advancing through preclinical studies and have already entered early-phase clinical trials.

However, most of these studies still rely on receptor architectures originally developed for CAR-T cells. While such constructs are functional in NK cells, they do not fully exploit NK-specific signaling pathways. Given the broad repertoire of activating receptors and adaptor protein domains that regulate NK cell responses, the development of NK-tailored CAR designs—through innovative combinations of extracellular, transmembrane, and intracellular domains—offers a rational strategy to improve therapeutic potency and specificity, thereby enhancing the overall efficacy of CAR-NK immunotherapy.

## 4. NK Cells Versus T Cells: Distinct Advantages for CAR-Based Immunotherapy

CAR T-cell therapy has transformed the landscape of cancer treatment, with several products gaining regulatory approval and demonstrating clinical benefit, including in patients with MM [36,37]. Nevertheless, there are notable limitations that challenge its broader application. Autologous CAR-T products require complex and costly manufacturing and may fail due to prior therapies, patient lymphopenia, or high tumor burden. In addition, the autologous production process takes valuable time, which can delay treatment for patients with aggressive disease. While universal allogeneic CAR-T approaches are under development, they necessitate extensive gene editing [38] to eliminate endogenous T-cell receptor signaling and mitigate the risk of GvHD, thereby increasing manufacturing complexity and raising safety concerns regarding genomic stability.

Current evidence indicates that clinical outcomes of allogeneic CAR-T-based therapies are encouraging but still lag behind the efficacy achieved with FDA-approved autologous CAR-T products [39]. For example, the first BCMA-directed CAR-T therapy, idecabtagene vicleucel (Abecma), approved in 2021, demonstrated an overall response rate of 72% and a stringent complete response rate of 28% in a pivotal study of 100 patients with relapsed/refractory MM [36]. Allogeneic CAR-T platforms have yet to reach similar efficacy benchmarks (NCT04093596). Ongoing efforts focused on optimizing cell engineering, enhancing persistence, and reducing alloreactivity are expected to improve their therapeutic durability and clinical impact.

In addition to logistical and manufacturing barriers, CAR-T therapy is often associated with serious adverse events. CRS and ICANS occur in the majority of patients treated with CAR-T products [40,41,42] and are driven by elevated levels of proinflammatory cytokines, including those secreted by activated T cells, such as IL-2, TNF-α, and IL-6 [43,44]. Other complications, including hemophagocytic lymphohistiocytosis [6], prolonged cytopenia [45,46], and GvHD in the allogeneic setting [47], further limit the safety profile of CAR-T therapy.

By contrast, CAR-NK cells offer several distinct advantages. First, NK cells do not rely on antigen recognition via MHC presentation, allowing them to exert cytotoxicity without prior antigen priming. Once modified with CARs, they retain their innate killing ability through germline-encoded activating receptors. This dual mechanism—CAR-dependent and CAR-independent cytotoxicity—provides a safeguard against antigen escape, a major limitation of CAR-T therapy [48,49]. CAR-T cells require relatively high levels of TAA expression for effective activation via their CARs [50,51], making them vulnerable to TAA downregulation or loss; CAR-NK cells can overcome this barrier through their intrinsic cytotoxic pathways. This capacity enables them to eliminate malignant cells even in the context of reduced or absent CAR target expression, underscoring their potential to mitigate one of the key challenges of CAR-T therapy. A notable example of CAR-NK engineering is FT576, a product designed for MM therapy. FT576 integrates a BCMA-targeting CAR with an engineered high-affinity, non-cleavable CD16 (hnCD16) receptor, enabling dual functionality: direct CAR-mediated recognition of BCMA-positive tumor cells and antibody-dependent cellular cytotoxicity (ADCC) in combination with therapeutic monoclonal antibodies. Importantly, preclinical evaluation demonstrated that FT576 exhibited BCMA targeting activity comparable to that achieved with primary BCMA-directed CAR-T cells, while additionally providing the potential for multiantigen targeting through its hnCD16-mediated mechanism [52]. This combinatorial design underscores the therapeutic promise of CAR-NK platforms to match or even expand upon the functional capabilities of CAR-T cells. Nonetheless, direct comparative studies of CAR-NK and CAR-T efficacy remain limited, particularly in the context of MM, highlighting the need for further systematic investigation.

Second, NK cells inherently carry a minimal risk of GvHD, enabling the development of allogeneic CAR-NK therapies [53,54,55]. Multiple cellular sources, including blood, stem and progenitor-cell-derived populations, and cell lines, can be exploited, improving accessibility and scalability compared with patient-derived CAR-T cells.

Despite their inherent safety in the allogeneic setting, a key challenge for CAR-NK therapies remains their limited in vivo persistence, partly due to immune rejection by the host. Unlike allogeneic CAR-T platforms, which require extensive gene editing to remove the endogenous T-cell receptor and prevent GvHD, allogeneic CAR-NK cells primarily need strategies to enhance survival rather than to ensure safety [56]. Recent studies have explored targeted modifications to prolong CAR-NK persistence, such as β2-microglobulin (B2M) knockout to reduce HLA-I expression and the co-expression of non-classical HLA molecules (e.g., HLA-E or HLA-G) to avoid host NK-cell recognition. Such strategies are conceptually analogous to those being developed for allogeneic CAR-T platforms but are generally simpler due to the absence of endogenous TCR-mediated alloreactivity in NK cells. These refinements aim to mitigate host-versus-graft rejection while preserving the simplicity and safety of NK-based platforms [57]. Additional approaches include deletion of adhesion ligands such as CD54 (ICAM-1) and CD58 (LFA-3) to reduce recognition and attack by host immune effectors [58], co-expression of IL-15 or membrane-anchored cytokines to boost survival and proliferation, and expression of anti-phagocytic “don’t-eat-me” signals like CD47 [59]. These combinatorial strategies have shown promise in preclinical and early clinical studies for improved in vivo durability.

Building on these engineering advances, several allogeneic CAR-NK platforms have already demonstrated clinical activity with favorable safety profiles, supporting the notion that NK-based therapies may offer reduced toxicity compared with CAR-T approaches. For instance, iPSC-derived CAR-NK products such as FT596 and FT576 have achieved objective responses in patients with relapsed or refractory lymphomas and MM, respectively, while exhibiting only low-grade CRS and no reported neurotoxicity [60,61]. These findings align with the intrinsic biology of NK cells, which predominantly secrete IFN-γ and GM-CSF [62] rather than high levels of IL-6 or TNF-α, cytokines strongly associated with cytokine storm and neurotoxicity. Supporting this, a clinical trial with cord-blood-derived CAR-NK cells for B-cell malignancies reported no increase in IL-6 or TNF-α levels above baseline after infusion [33]. Similarly, studies employing CAR-engineered invariant natural killer T cells (CAR iNKT) in MM models demonstrated significantly lower IL-6 induction in vitro compared with CAR-T counterparts [63]. Together, these data highlight that, even with limited in vivo persistence, allogeneic CAR-NK cells can mediate meaningful antitumor responses with a more favorable safety profile and may serve as a readily deployable alternative to autologous CAR-T therapy.

Finally, cost is another important factor favoring NK-based therapies. A single infusion of an approved CAR T-cell product currently amounts to several hundred thousand dollars [64], and when additional pre- and peri-infusion healthcare expenses are taken into account—for example, exceeding $150,000 in patients with relapsed or refractory MM [65]—the overall economic burden becomes even greater. The possibility of standardized, allogeneic CAR-NK products promises a more cost-effective and broadly accessible immunotherapy approach.

Taken together, CAR-NK cells combine innate safety, accessibility, and a unique dual killing mechanism, distinguishing them from CAR-T cells. While comparative antitumor efficacy remains an active area of investigation—with some studies favoring CAR-T and others suggesting benefits for CAR-NK in specific contexts—the accumulating evidence supports CAR-NK as a promising alternative platform for next-generation cellular immunotherapy.

## 5. Sources for CAR-NK-Cell-Based Adoptive Immunotherapy in Multiple Myeloma

To date, current clinical applications of NK cell therapy for MM include both autologous and allogeneic NK-cell-based approaches. A study by Nahi et al. demonstrated the safety and potential efficacy of repeated infusions of activated and expanded autologous NK cells following autologous stem cell transplantation in patients with MM [66]. However, in patients with MM, NK cells frequently exhibit a dysfunctional phenotype, characterized by altered transcriptional profiles and markedly reduced cytotoxic capacity [67,68,69]. The majority of clinical trials utilizing NK cells require high infusion doses, commonly between 5 × 10^6^ and 1 × 10^8^ NK cells per kilogram of body weight [70]. At the same time, the production of autologous NK cells remains technically challenging and resource-intensive, often complicated by the limited availability of patient-derived cells for ex vivo expansion and genetic engineering. Such functional deficiencies significantly constrain the therapeutic potential of autologous NK-cell-based approaches.

**Figure 2:**
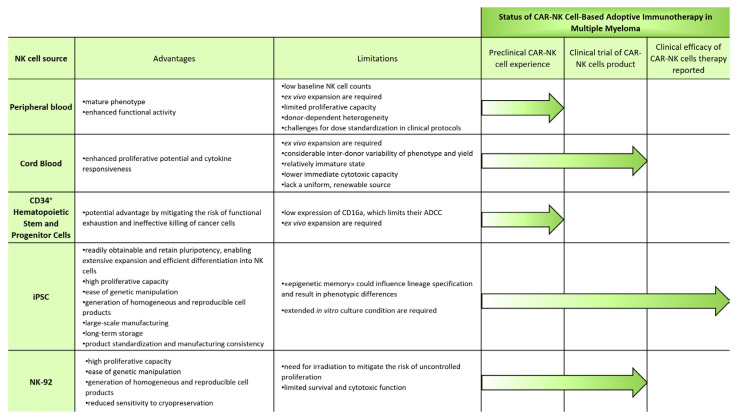
Different sources of CAR-NK cells for the treatment of multiple myeloma.

The rationale for utilizing allogeneic NK cells has initially stemmed from insights into the molecular specificity of NK cell recognition, particularly their ability to mediate missing-self reactivity [18]. Due to a low risk of GvHD induction, allogeneic NK cells have emerged as the preferred platform in contemporary NK cell therapy programs, thereby bypassing the limitations inherent to autologous strategies [71,72]. Moreover, NK cells from healthy allogeneic donors may exhibit superior functionality compared to autologous NK cells obtained from heavily pretreated patients, lymphodepleting therapy in particular. To address the restricted availability of NK cells from individual donors, several large-scale expansion strategies are under investigation, with the theoretical potential to generate sufficient therapeutic doses to treat thousands of patients from a single source. Within this context, three major categories of allogeneic NK cell sources have been explored, including blood, stem- and progenitor-cell-derived populations, and cell lines [73] (Figure 2). PB [74] and CB [75,76] have long served as primary sources for NK cell expansion. In parallel, robust protocols have been developed to produce NK cells from embryonic stem cells (ESCs) [77] and iPSCs [78,79], as well as from CD34-expressing hematopoietic stem and progenitor cells (HSPCs) derived from umbilical CB and placenta blood [80]. An alternative strategy involves the use of NK-92, an immortalized NK cell line that can be irradiated prior to administration to prevent uncontrolled proliferation in vivo [81]. These expansion and manufacturing approaches enable the production of multiple doses of allogeneic NK cells, which are relatively short-lived and thus may require repeated administration. Notably, these cells can be used fresh or cryopreserved after being prepared in advance, offering a flexible “off-the-shelf” therapeutic platform for clinical application in MM and solving the one-donor, one-patient limitation.

All of the aforementioned allogeneic NK cell sources are capable of generating therapeutically relevant cell doses and amenable to CAR engineering. At the same time, each source presents distinct advantages and limitations, and may exhibit unique scalability, transcriptional landscapes, phenotypic characteristics, and functional profiles. As of now, ten clinical trials exploring CAR-NK cell therapy for MM have been registered. Among them, at least five are based on allogeneic NK cells: two trials relied on CB-derived NK cells, one study reported the use of iPSC-derived NK cells, another utilized NK-92-derived cells, and one trial involved NK cells of unspecified origin. For the remaining studies, the source of NK cells has not been made publicly available. Of note, among these ten registered clinical trials, seven are currently ongoing, while the current status of the remaining three has not been updated in official registries, as summarized in Table 1. This distribution underscores the dynamic and still-evolving landscape of CAR-NK-cell-based approaches in MM.

### 5.1. Blood

NK cells can be isolated from apheresis products of PB (PB-NK cells) or from CB (CB-NK cells) using cell sorting techniques or immunomagnetic cell separation platforms. Enrichment of highly pure NK cell populations is essential to optimize subsequent expansion and to minimize impurities in the final cell product. This is particularly important for ensuring patient safety by reducing the risk of residual allogeneic T cells, which could induce GvHD.

#### 5.1.1. Peripheral Blood

PB represents a readily accessible and clinically valuable source of mature NK cells for adoptive immunotherapy. Mature phenotype of PB-NK cells enhances their functional activity but limits their proliferative capacity [82]. PB-NK cells are classically defined by their expression profile—CD3^−^, CD14^−^, CD19^−^, CD56^+^, CD16^+/−^ [83]—and can be further divided into two major phenotypic subsets: CD56^bright^ and CD56^dim^ cells, with the latter being predominant in circulation (approximately 90%) [84]. NK cells typically represent 5–10% of circulating lymphoid cells in PB [85], although their frequency can vary substantially between individuals, with reported values ranging from 0% to 60% CD56^+^CD3^−^ cells in certain cohorts [86]. Despite their relatively short lifespan—estimated at around two weeks [87]—adult individuals typically harbor more than 2 billion NK cells at any given time [88]. In fact, PB-NK cells were instrumental in the early development of CAR-NK cell therapies. The first successful delivery of a CAR construct into NK cells was conducted using PB-derived NK cells in 2005 by Dario Campana’s group [89].

The efforts are currently underway to use PB-NK cells in the development of CAR-engineered therapeutic platforms targeting MM. In a study by Ng et al. PB-NK cells were expanded by co-culturing with feeder cell line K562 that express membrane-bound IL-15, membrane bound IL-21, and CD137L. After stimulation for 14 days, PB-NK cells with a final purity of >90% were collected and used for mRNA-transfection with downstream experiments [90]. A similar approach enabled an approximate 4000-fold rapid expansion of high-purity PB-NK cells [24]. At the same time, PB-NK cells display notable phenotypic and functional variability among donors, which appears to be driven by both genetic factors and environmental stimuli. Such donor-dependent heterogeneity along with low baseline NK cell counts and limited proliferative capacity pose challenges for dose standardization in clinical protocols, and hence generating consistent and scalable therapeutic products. These limitations are especially relevant in the context of CAR-NK cell therapy in MM, where genetic modification and ex vivo expansion are required.

Nevertheless, several preclinical studies are investigating different strategies for CAR-engineered PB-derived NK cells in MM models. These include double-modified NK cells designed to mitigate the risk of antigen escape [90], as well as non-antibody approach that exploit CAR constructs based on the NKG2D receptor [23]. Furthermore, there is information suggesting that BCMA/GPRC5D dual-targeted CAR-NK cells, which have demonstrated efficacy in both in vivo and in vitro models of MM [91], are derived from PB [92]. At present, publicly available registries do not provide conclusive information indicating that clinical trials have been initiated with PB-derived CAR-NK cells for the treatment of MM.

#### 5.1.2. Cord Blood

NK cells isolated from CB, which is readily available from global CB banks, represent another potential source material for CAR-NK cell therapies in MM. In contrast PB on average 30% of the total lymphocytes in CB are NK cells [93]. The phenotypic profile of CB-NK cells is characterized by elevated expression of CD56^bright^, NKG2A, CD94, c-kit (CD117), Trail, CD62L, and CD27 [94], along with reduced levels of KIRs, NKG2C, NKp46, and DNAM-1 [95]. At the transcriptomic level, CB-NK cells exhibit lower expression of maturation-associated markers such as T-bet, eomesodermin, perforin, granzyme A, and granzyme B [94]. This phenotypic and transcriptional profile reflects the relatively immature state of CB-NK cells, which is associated with lower immediate cytotoxic capacity but enhanced proliferative potential and cytokine responsiveness. These features make CB-NK cells a suitable candidate for ex vivo expansion and genetic engineering in adoptive immunotherapy in MM.

CB-NK cells can be effectively expanded and driven toward a more mature profile through cytokine stimulation. Compared to PB-NK cells, CB-NK cells are less responsive to IL-2, and 5 times more IL-2 is required to activate them [95], which is attributed to lower expression of IL-2 receptors and reduced induction of the p-STAT5 pathway [96]. However, stimulation of CB-NK cells with a combination of IL-15 and IL-18 resulted in the most robust proliferative response and increased secretion of IFN-γ and TNF-α, whereas activation with a combination of IL-15 and IL-2 promoted enhanced cytotoxicity [96]. Additionally the expansion of CB-NK cells can be enhanced through co-culture with feeder cells, such as genetically modified K562 expressing membrane-bound IL-21, 4-1BB ligand, and CD48, resulting in a proliferation rate increase exceeding 900-fold [97].

Preclinical studies have demonstrated the feasibility and therapeutic potential of using CB-NK cells to develop CAR-engineered platforms targeting MM. Castellano et al. aimed to develop a cord-blood-derived CAR-NK product using CRISPR/Cas9 technology to disrupt the expression of NK receptors involved in some dominant immunosuppressive signals in the tumor microenvironment [98]. In 2023, Lin et al. reported the generation and evaluation of clinical-grade CAR-NK cell products derived from umbilical CB [99]. Based on these findings, a Phase I/II clinical trial (NCT05092451) was initiated and is currently recruiting participants. Another clinical trial is also known to be using CAR-engineered NK cells derived from umbilical and CB in patients with relapsed or refractory MM (NCT05008536). At the same time, as with PB-NK cells, limitations of this source of NK cells for CAR-NK cell therapy in MM are still that their phenotype and yield exhibit considerable inter-donor variability, and they lack a uniform, renewable source.

### 5.2. Stem and Progenitor Cells

While human CD56^+^ NK cells isolated from blood can be expanded ex vivo, NK cells can also be derived from pluripotent stem cells, as well as from CD34-expressing HSPCs, upon differentiation and then can be expanded in a stepwise fashion. This approach enables the generation of a more homogeneous and well-defined NK cell product compared with NK cells that develop in utero.

#### 5.2.1. CD34^+^ Hematopoietic Stem and Progenitor Cells

NK cells can be differentiated in vitro from CD34^+^ HSPCs derived from umbilical cord and placenta blood. HSPCs are typically isolated through immunomagnetic positive selection targeting the CD34 surface antigen, using a process similar to that employed for leukapheresis-derived products [100].

NK cells generated through this approach are characterized by elevated expression of CD56, NKG2A, and CD94, along with variable levels of KIRs, and therefore exhibit a less mature phenotype compared to PB-NK cells [101]. As was reported, this fact offers a potential advantage by mitigating the risk of functional exhaustion and ineffective killing of cancer cells after infusion in the recipient that typically affects more mature PB- and CB-derived NK cells after extended cytokine-driven expansion [80]. At the same time although these CD56^+^ cells demonstrate cytotoxic activity against MM cell line [102], they exhibit low expression of CD16a (FcγRIIIA) compared to CB-NK cells, which limits their ADCC [103,104].

Celularity Incorporated (USA) has developed a large-scale GMP-compliant expansion and differentiation process of UCB-CD34^+^ cells into PNK-007 NK cells. PNK-007 is the first fully allogeneic CD34^+^ derived NK cell product in MM clinical trials (NCT02955550). A single infusion of PNK-007 at doses of up to 30 × 10^6^ cells/kg, administered with or without rhIL-2, was well tolerated in the post-autologous stem cell transplantation setting [105]. Translational findings from a Phase I study further demonstrated that administration of PNK-007 at doses of up to 3 × 10^7^ cells/kg, given either 14 or 7 days post-transplant, did not adversely affect engraftment or immune reconstitution [106]. Later, in 2020, a study with CYNK-001, which is cryopreserved successor of the previous fresh UCB-CD34 product, PNK-007, was also registered (NCT04309084).

With respect to CAR-NK cell therapy for MM based on CD34^+^ HSPCs, this strategy has not yet been translated into clinical practice. Despite significant advancements at the preclinical stage and in the development of candidate product—such as PNK-CAR38, which has demonstrated potent cytotoxic activity against MM cell line in vitro without evidence of on-target, off-tumor toxicity against healthy donor-derived cells [102]—these therapies have not yet been evaluated in patients with MM.

#### 5.2.2. iPSCs

As previously noted, human ESCs can be used to generate a homogeneous population of functional NK cells exhibiting strong cytotoxic activity in vitro and potent antitumor efficacy in vivo [77,78]. At the same time, in recent years, the most significant advances in the development of CAR-NK-cell-based therapies for MM have been achieved through the use of the iPSC platform. This may be attributed to the fact that, unlike clinical trials targeting degenerative eye diseases, neurodegenerative disorders, and type 1 diabetes, studies investigating immunotherapies for malignancies are, in principle, predominantly based on iPSC-derived approaches [107].

Derived from reprogrammed somatic cells, such as fibroblasts or PB cells, as a rule, iPSCs are readily obtainable and retain pluripotency, enabling extensive expansion and efficient differentiation into NK cells. The establishment of iPSC master cell banks ensures a continuous supply of genetically uniform donor material [108] and provides opportunities for product standardization and manufacturing consistency [109].

NK cells derived from iPSCs express key NK cell markers such as CD56, CD117, CD94, NKp46 [110], as well as DNAM-1, CD69, NKG2A and NKG2D, NKp44, NKp30 [111]. Goldenson et al. found that iPSCs-derived NK cells exhibit heterogeneous KIR expression profiles, with some populations displaying high levels of KIRs (KIR2DL3, KIR2DL1, KIR2DL2, and KIR2DL1), whereas others show minimal or absent KIR expression. At the same time, despite variations in KIR expression, both iPSC-KIR^+^ and iPSC-KIR^−^ NK cell populations demonstrate comparable cytotoxic activity overall [103].

A potential limitation of iPSC-derived NK cells lies in the fact that iPSCs may retain DNA methylation patterns reflective of their somatic cell of origin. This “epigenetic memory” could influence lineage specification and result in phenotypic differences compared to the donor-derived cells, highlighting the need for careful consideration when employing iPSC-based platforms [112]. It was shown that blood-derived iPSCs exhibited more robust in vitro hematopoiesis compared to their neural-derived counterparts [113]. Nevertheless, an increasing number of genetically engineered iPSC-NK cell candidates have demonstrated promise in preclinical studies, with several advancing into early-phase clinical trials.

Woan et al. developed a clonal iPSC line incorporating triple-gene edits and subsequently differentiated it into NK cells, designated as iADAPT. iADAPT NK cell product demonstrates in vivo persistence without the need for exogenous cytokine support, exhibits potent antitumor activity, and can be effectively combined with daratumumab to enhance the cytotoxic elimination MM cells in both in vitro and in vivo models [114]. Collectively, these preclinical findings have provided a strong rationale for advancing iADAPT NK cells to phase I clinical trials for the treatment of patients with advanced malignancies (NCT04614636) sponsored by Fate Therapeutics.

Recent research has described the development of iPSC-derived NK cells with four integrated functional edits designed to dual-target MM by incorporating an NK cell-optimized BCMA-specific CAR and a high-affinity, non-cleavable CD16 (hnCD16) receptor, termed as iDuo-MM CAR-NK cells. Across multiple preclinical models, including xenogeneic adoptive transfer systems, these cells have consistently exhibited sustained antitumor activity against MM and enhanced ADCC in the presence of therapeutic anti-CD38 antibodies [115,116]. These findings strongly support the clinical translation of this platform (FT576) as a promising therapeutic strategy for the treatment of MM (NCT05182073).

### 5.3. NK Cell Lines

To overcome the challenges associated with primary NK cell sources—including donor-to-donor variability, difficulties in large-scale purification and expansion, and resistance to genetic modification—researchers have turned to the development of immortalized NK cell lines as a reliable alternative for adoptive immunotherapy. These cell lines represent homogeneous NK cell populations that can be readily maintained and expanded in vitro, providing a consistent and scalable source of therapeutic NK cells. To date, at least ten NK cell lines have been established, including NK-92, YT (also referred to as YT-S), KHYG-1, NK3.3, NK-YS, NKL, NKG, SNK-6, and IMC-1. Among these, NK-92, established from a patient with malignant non-Hodgkin’s lymphoma, remains the only line that closely resembles primary blood-derived NK cells and exhibits strong cytotoxic and cytostatic activity. Consequently, NK-92 has been successfully adapted for CAR NK-cell-based therapy in hematologic malignancies, including MM, with both preclinical studies and clinical trials already confirming its therapeutic promise.

#### NK-92

According to the American Type Culture Collection (ATCC, Manassas, VA, USA), NK-92 cells display a phenotypic profile characterized by the expression of CD2, CD7, CD11a, CD28, CD45, CD54, and CD56^bright^, while lacking surface expression of CD1, CD3, CD4, CD5, CD8, CD10, CD14, CD16, CD19, CD20, CD23, CD34, and HLA-DR [117]. The broad cytotoxic potential of NK-92 cells is attributed to their expression of the full repertoire of currently identified activating receptors [118], while exhibiting only a limited set of inhibitory receptors, including LIR/ILT, CD94/NKG2A, and KIR2DL4 [119,120].

In addition to their lack of most inhibitory KIRs, which reduces the likelihood of functional inhibition, NK-92 cells also do not express CD16a (FcγRIIIa), a critical receptor responsible for mediating ADCC and acting as a major activator of NK cell cytotoxicity. Nevertheless, genetic engineering approaches have successfully introduced high-affinity recombinant IgG Fc receptor (FcγR) [121] or reactivated endogenous CD16 expression [122] in NK-92 cells, thereby enhancing their antitumor efficacy in combination with various monoclonal antibodies.

Irradiation, while necessary to mitigate the risk of uncontrolled proliferation, has a detrimental impact on NK-92 cell survival and cytotoxic function, rendering these cells highly susceptible to Fas-mediated apoptosis and killing by primary blood NK cells. Consequently, innovative strategies are indicated to replace irradiation as an antiproliferative measure, including genetic disruption of Fas and other NK cell activation ligands, with the goal of enhancing the persistence and therapeutic efficacy of NK-92-derived cell products [123].

A review of the literature indicates that, to date, three preclinical studies have evaluated CAR-NK cell therapy targeting MM utilizing the NK-92 cell line as an effector platform [124,125,126]. In addition, a multicenter, phase I dose-escalation clinical trial was registered China to evaluate a second generation anti-BCMA CAR construct utilizing the NK-92 cell line (NCT03940833).

## 6. Targets for CAR-NK Cell Therapy in Multiple Myeloma

**Figure 3:**
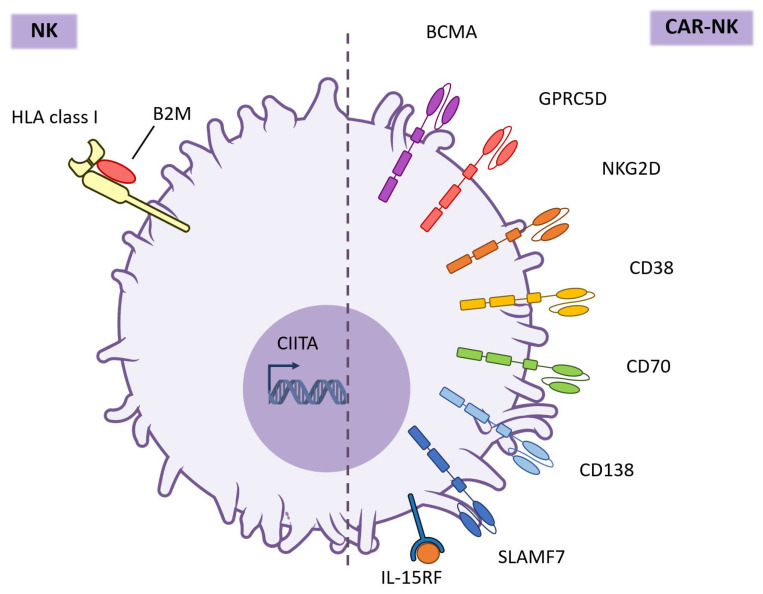
Current targets for CAR-NK Cell therapy in Multiple Myeloma.

Given the heterogeneity of available NK cell sources and their distinct biological properties, the design of CAR constructs must be carefully aligned not only with the expansion potential and safety profile of the selected NK population but also with the therapeutic target in MM. While the optimization of NK-specific signaling domains represents one critical step toward maximizing efficacy, an equally important consideration is the choice of TAA. In MM, where clonal evolution and antigen escape pose major challenges, the rational selection of appropriate targets such as BCMA, GPRC5D, NKG2D, CD38, CD70, CD138, and SLAMF7 has become a central focus in advancing CAR-NK therapy (Figure 3).

### 6.1. BCMA

BCMA (also known as TNFRSF17) is a well-validated target for MM therapy due to its highly restricted expression on normal and malignant plasma cells. This selectivity minimizes the risk of off-tumor toxicity while ensuring broad coverage across MM clones. However, BCMA shedding by γ-secretase can decrease surface antigen density [127] and lead to therapeutic resistance, motivating the development of strategies such as γ-secretase inhibition and dual-targeting constructs to enhance antigen availability and durability of response.

Preclinical studies have consistently demonstrated that NK cells engineered with anti-BCMA CARs exert potent cytotoxic effects against MM cells in vitro and in vivo. Two recent series of studies used NK cell platforms derived from the NK-92 cell line and from iPSC-derived NK cells. NK-92-based platforms expressing anti-BCMA CARs (including constructs that co-express pro-apoptotic payloads such as soluble TRAIL) showed enhanced killing of MM cell lines and primary samples, increased secretion of IFN-γ and granzyme B, and significant antitumor efficacy in xenograft models [25,128]. When combined with agents such as bortezomib or γ-secretase inhibitors, these CAR-NK cells exhibited synergistic effects, highlighting the translational potential of rational combination approaches [25,128].

A major advancement has been the development of iPSC-derived CAR-NK products, exemplified by the FT576 platform. This product integrates multiple edits—an optimized anti-BCMA CAR, a high-affinity non-cleavable CD16 receptor to augment ADCC, a membrane-bound IL-15/IL-15R fusion to support in vivo persistence, and CD38 knockout to prevent fratricide during combination with daratumumab. These iNK cells with four integrated functional edits demonstrated sustained, cytokine-independent antitumor activity in MM xenograft models, supporting an “off-the-shelf” allogeneic approach with enhanced functionality and persistence [61,115,116]. Complementary CRISPR/Cas9-based multiplex gene editing strategies have been explored to remove inhibitory receptors and improve metabolic fitness, aiming to further potentiate NK-cell effector function within the suppressive tumor microenvironment [98].

Preclinical activity has been assessed using cytotoxicity assays, cytokine profiling, serial restimulation experiments, and both localized and disseminated xenograft models. The absence of uncontrolled cytokine release or off-target cytotoxicity across studies reinforces the favorable safety profile of CAR-NK platforms.

Translational progress is reflected in several ongoing early-phase clinical trials, including the evaluation of FT576 in the Phase I study (NCT05182073). Twelve patients received treatment following a three-dose schedule, administered either as monotherapy or in combination with CD38-targeted monoclonal antibody therapy. Patients were treated with doses of 1 × 10^9^ cells (*n* = 6) or 2.5 × 10^9^ cells (*n* = 6) per infusion. Interim clinical reports have shown biological activity in low-dose cohorts with decreases in serum BCMA levels, alongside a complete absence of CRS, ICANS, or GvHD, supporting the favorable safety profile predicted from preclinical studies. Among the six heavily pretreated patients who received FT576 at a dose of 1 × 10^9^ cells per infusion, five (83%) achieved a clinical response. Notably, two penta-exposed patients treated with FT576 as monotherapy achieved very good partial responses [129]. Additional trials (NCT05008536, NCT03940833, NCT05652530, NCT06045091, and NCT06242249) are investigating diverse CAR constructs and NK-cell sources, including umbilical- and cord-derived and NK-92-based products, primarily focusing on safety, persistence, and preliminary efficacy in relapsed/refractory MM.

Despite encouraging progress, several challenges remain. BCMA loss and shedding continue to drive escape mechanisms, and the persistence of CAR-NK cells in vivo remains a key area for optimization. Advances in NK-specific CAR design, cytokine engineering, and multiplex genomic editing hold promise for improving potency, durability, and resistance to antigen escape. Collectively, current data position BCMA as the most advanced and clinically validated target for CAR-NK therapy in MM, bridging potent anti-myeloma activity with a favorable safety profile that may ultimately complement or extend the success of BCMA-directed CAR-T approaches.

### 6.2. BCMA-Based Dual-Target

Despite the significant progress achieved with BCMA-directed immunotherapies, including CAR-T and CAR-NK products, therapeutic resistance remains a major clinical challenge. Mechanisms such as antigen loss, heterogeneous BCMA expression, and soluble BCMA-mediated neutralization can lead to relapse following initially successful responses. To overcome these limitations, researchers have explored dual-targeting CAR-NK cell strategies, integrating BCMA recognition with additional TAA to broaden tumor coverage, enhance trafficking, and reduce antigen escape. This section discusses the main BCMA-based dual-target CAR-NK approaches reported to date, each designed with distinct biological rationales and engineering concepts.

#### 6.2.1. BCMA and CD19

The combination of BCMA and CD19 targets aims to address tumor heterogeneity across different stages of B-cell maturation. While BCMA is highly expressed on mature plasma cells, CD19 is retained on earlier B-lineage cells and in certain myeloma stem-like subsets [130]. Thus, BCMA—CD19 dual-target CAR-NK cells were developed to simultaneously eradicate plasma cells and precursor populations, potentially preventing relapse arising from residual CD19^+^ myeloma progenitors.

A representative study by Roex et al. demonstrated that dual BCMA/CD19 CAR-NK cells effectively lysed tumor B-cell lines and primary patients samples while maintaining selective recognition of malignant cells, even at low effector to target ratios [131]. Dual-CAR NK-92 cells have been shown to retain their functional activity following gamma irradiation, supporting their potential as an “off-the-shelf” platform for clinical application. The CAR constructs in this model contained scFvs recognizing BCMA and CD19, and utilized CD8α hinge and transmembrane regions coupled to 4-1BB and CD3ζ signaling domains, optimized for NK activation [131].

#### 6.2.2. BCMA and GPRC5D

GPRC5D, a G protein–coupled receptor of unknown physiological ligand, has recently gained prominence as a promising antigen for immunotherapy in MM [132]. Its expression is largely restricted to malignant plasma cells, with minimal presence in normal tissues apart from limited hair follicle keratinocytes, suggesting a favorable safety window. Importantly, GPRC5D expression often persists in MM clones that have lost or downregulated BCMA, making it an attractive complementary or independent therapeutic target. Thus, combining BCMA and GPRC5D recognition is a rational strategy to mitigate antigen escape and maintain efficacy in tumors with heterogeneous antigen profiles.

Recent preclinical and early clinical investigations (NCT06594211), including those presented at American Society of Hematology (ASH) 2022 and American Association for Cancer Research 2023, explored allogeneic BCMA/GPRC5D dual-target CAR-NK constructs [91,133]. These CAR-NKs employed tandem or bicistronic architectures enabling recognition of both targets. Early preclinical data demonstrated potent cytotoxicity against diverse MM cell lines regardless of BCMA or GPRC5D expression levels, and improved disease control in xenograft models compared to single-antigen CAR-NKs.

A clinical program (NCT06594211) is now evaluating this dual-target CAR-NK therapy in patients with relapsed/refractory MM. The rationale for this program underscores redundant antigen targeting to prevent clonal escape and enhanced durability through combined antigen coverage.

#### 6.2.3. BCMA and CXCR4

Incorporation of CXCR4 into CAR-NK constructs does not represent a second antigenic target but rather a homing modification to enhance NK-cell migration toward the bone marrow niche—where MM cells predominantly reside. Since NK cells often exhibit limited trafficking to the marrow microenvironment, CXCR4 co-expression restores their responsiveness to stromal CXCL12 gradients.

Ng et al. engineered BCMA-CAR PB-NK cells co-expressing CXCR4 and demonstrated improved in vivo tumor localization and prolonged survival in MM-bearing mice compared to unmodified BCMA-CAR NK cells [90]. This strategy effectively addressed a key barrier to NK-based immunotherapy: insufficient tumor homing. The CAR construct itself was a conventional second-generation design with anti-BCMA scFv–CD8α hinge–CD8α TMD–CD3ζ (or DAP12) signaling domain, while the additional CXCR4 transgene provided chemotactic advantage without altering target specificity. This “homing-enhanced” design represents a promising direction for increasing the translational efficacy of BCMA CAR-NKs.

BCMA-based dual-target strategies illustrate the logical progression of CAR-NK therapy from single-antigen precision to multi-dimensional tumor recognition. Each secondary component—CD19, GPRC5D or CXCR4—addresses a distinct biological challenge: CD19 extends recognition to early progenitor clones; GPRC5D mitigates BCMA heterogeneity and escape; CXCR4 improves bone marrow homing.

Collectively, these approaches demonstrate how rational CAR design can integrate antigen coverage, localization, and functional persistence to optimize therapeutic outcomes. As the field advances, future constructs may incorporate logic-gated or tandem CAR architectures and gene edits that dynamically modulate NK activation in response to complex MM microenvironments.

### 6.3. GPRC5D

A recent study presented at the ASH 2023 [134] described the development and preclinical evaluation of human iPSC-derived CAR-NK cells targeting GPRC5D. These NK cells were engineered to express a GPRC5D-specific CAR optimized for NK signaling using a piggybac transposon system. In vitro assays demonstrated potent antigen-specific cytotoxic activity against BCMA^+^/GPRC5D^+^ MM cell line (NCI-H929), showing an overwhelming advantage of the engineered CAR-NK cells, with approximately 90% tumor cell lysis compared to minimal activity observed in CB-NK cells (no detectable killing) and wild-type iPSC-derived NK cells (around 10% killing rate). Moreover, cryopreserved GPRC5D targeting CAR-engineered NK cells retained comparable antigen-specific cytotoxicity in vitro and demonstrated the capacity to reduce tumor burden in an antigen-dependent manner in the xenograft model.

These results provide strong preclinical validation for GPRC5D as a standalone CAR-NK target in MM and complement emerging dual-target strategies that include BCMA. The consistent efficacy across MM models highlight GPRC5D’s translational promise for future clinical development of allogeneic CAR-NK therapies aimed at overcoming antigen escape and improving long-term disease control.

### 6.4. NKG2D

NKG2D is an activating receptor broadly expressed on NK cells and subsets of T cells that recognizes stress-induced ligands such as MICA, MICB, and ULBP family proteins, frequently upregulated on malignant plasma cells but largely absent from normal tissues. This ligand multiplicity makes NKG2D an attractive target for CAR engineering in MM, offering the potential to overcome antigen heterogeneity and escape that limit single-antigen approaches. Preclinical studies have demonstrated that NKG2D-based CAR-NK cells can efficiently recognize and lyse MM cells in vitro and control tumor progression in vivo [23,135].

Leivas and colleagues generated NKG2D-CAR-modified NK cells derived from patient PB, using a construct in which the NKG2D ectodomain was fused to intracellular 4-1BB and CD3ζ signaling domains [135]. These CAR-NK cells exhibited robust cytotoxicity against various MM cell lines and primary MM plasma cells, while sparing healthy cells. In xenograft models, treatment significantly reduced tumor burden and improved survival without evidence of toxicity. The same group’s earlier work presented at ASH 2018 confirmed efficient gene transfer, stable CAR expression, and selective killing of BCMA-positive and BCMA-negative MM populations, highlighting the advantage of NKG2D CARs in addressing clonal diversity [23]. A comparative analysis using NK-92 cells further supported these findings, showing that NKG2D CAR-NK cells achieved comparable or superior anti-myeloma activity relative to BCMA-CAR constructs, reflecting their broader recognition of stress ligands expressed on tumor cells [126].

Safety assessment in preclinical models revealed minimal off-target reactivity and no genomic instability following NK cell expansion, indicating a favorable profile for clinical translation. The first clinical trial investigating NKG2D-CAR-NK therapy in relapsed/refractory MM is currently underway, aiming to evaluate safety, tolerability, and early efficacy endpoints (NCT06379451). Although detailed CAR construct features have not been publicly disclosed, existing preclinical data strongly support the translational potential of this approach.

Overall, NKG2D-directed CAR-NK cells represent a promising strategy that broadens target coverage and may mitigate the challenge of antigen escape in MM. Their favorable safety profile, together with evidence of potent anti-myeloma activity, low cytotoxicity against healthy cells (lung, PBMCs), with a basal expression of NKG2D ligands, and absence of GvHD in preclinical models, positions NKG2D as a compelling complement to lineage-specific targets such as BCMA. Ongoing clinical evaluation will determine whether the pleiotropic ligand engagement of NKG2D can be harnessed effectively and safely in patients with MM.

### 6.5. CD38

CD38 is a transmembrane glycoprotein widely expressed on malignant plasma cells in MM, as well as on various hematopoietic and non-hematopoietic cell types, which introduces both opportunities and challenges for targeted immunotherapy. While its broad expression supports robust antigen accessibility, it also raises concerns about on-target, off-tumor cytotoxicity. Nevertheless, the clinical success of monoclonal antibodies such as daratumumab and isatuximab [136,137] has validated CD38 as a therapeutically actionable target and encouraged exploration of CD38-directed cellular therapies.

Preclinical studies have demonstrated that NK cells engineered with anti-CD38 CARs can effectively recognize and eliminate MM cells while maintaining an acceptable safety profile through the use of optimized receptor design and regulated expression. One of the earliest and most detailed reports used CD34^+^ HSPCs-derived allogeneic NK cells transduced with a retroviral vector carrying second generation CD38-specific CAR (PNK-CAR38 cells), which showed significant cytotoxic activity against MM cell lines both in vitro and in a disseminated lymphoma murine xenograft model [102].

Building on these findings, more recent work has developed NK-92-derived and PB-derived CAR-NK platforms incorporating CD38-directed receptor. PB-NK cells expressing anti-CD38 CAR demonstrated potent anti-myeloma cytotoxicity while exhibiting controlled activation to minimize fratricide and off-target toxicity [138].

An emerging strategy further expands the scope of CD38 targeting by combining it with other antigens to prevent immune escape. A dual-target construct co-recognizing GPRC5D and CD38 has been developed in the FT555 platform—a multiplex-engineered iPSC-derived CAR-NK product that integrates an anti-GPRC5D CAR, a second anti-CD38 CAR, a IL-15/IL-15 receptor fusion protein for cytokine-independent persistence, and a high-affinity CD16 receptor for enhanced ADCC. Preclinical data have shown that this dual-target approach preserves robust killing of MM cells with heterogeneous antigen expression, mitigating the risk of single-antigen loss and supporting a path toward more durable responses. The FT555-mediated antitumor activity further enhances when combined with daratumumab, resulting in deeper tumor growth inhibition and improved survival [139].

Together, these findings position CD38 as a valuable yet complex target for CAR-NK therapy in MM. While antigen expression on the surface of cells of myeloid and lymphoid lineage and cells of nonhematopoietic origin remains a potential limitation, the evolution of NK-specific CAR constructs, controlled signaling designs, and multiplex antigen targeting strategies—exemplified by the GPRC5D + CD38 dual CAR-NK platform—may enable effective and safe exploitation of CD38-directed immunotherapy in future clinical applications.

### 6.6. CD70

CD70, a member of the tumor necrosis factor family, is a type II transmembrane protein that interacts with its receptor CD27 to regulate lymphocyte activation and survival. Under physiological conditions, CD70 expression is transient and tightly restricted to activated immune cells. In MM, however, CD70 is aberrantly and persistently expressed on malignant plasma cells, while CD27 may be downregulated during disease progression, suggesting that CD70 signaling contributes to tumor proliferation, immune evasion, and resistance to apoptosis. This expression pattern, together with its limited presence on normal tissues, makes CD70 an attractive target for cellular immunotherapy in MM.

Recent preclinical work published in Blood [99] has explored the feasibility of CD70-directed CAR-NK cell therapy. CD70 expression was detected in all evaluated patients (10/10) who had relapsed following BCMA-targeted treatments, indicating its promise as an alternative antigen in cases of BCMA therapy resistance. CD70-specific CAR-NK cells demonstrated potent cytolytic activity against CD70^+^ MM cell lines, while showing the same cytotoxicity toward the CD70^−^ H929 myeloma line as non-engineered NK cells, confirming their antigen specificity. In a murine MM model, CD70 CAR-NK cells achieved superior tumor control and significantly prolonged survival, underscoring their therapeutic potential in relapsed or refractory MM.

Building on these findings, a first-in-human Phase I/II clinical trial (NCT05092451) is currently recruiting, which aims to evaluate the safety and preliminary efficacy of allogeneic CB-derived CD70-directed CAR-NK cells in patients with relapsed or refractory MM. This trial represents one of the earliest clinical efforts to assess CD70 as a viable NK-based therapeutic target in plasma cell malignancies. The study design includes dose escalation with multiple administration cycles, monitoring for CRS, ICANS, and GvHD.

Altogether, these data highlight CD70 as a promising and biologically rational target for CAR-NK therapy in MM. The combination of restricted antigen expression demonstrated in vivo efficacy, and early clinical translation supports further development of CD70 CAR constructs, particularly those incorporating NK-optimized signaling motifs to maximize cytotoxicity while minimizing off-tumor reactivity. Continued refinement of CAR architecture and exploration of dual-target strategies may further enhance the clinical potential of CD70-directed CAR-NK approaches in MM.

### 6.7. CD138

CD138 (syndecan-1) is a heparan sulfate proteoglycan abundantly expressed on the surface of normal and malignant plasma cells, playing a key role in cell adhesion, proliferation, and interaction with the bone marrow microenvironment. In MM, CD138 expression is not only a diagnostic hallmark but also contributes to disease pathogenesis by mediating tumor–stroma interactions and protecting myeloma cells from apoptosis. Its restricted expression in terminally differentiated plasma cells and minimal presence on other hematopoietic cells make CD138 an appealing therapeutic target for cellular immunotherapy in MM [40,140,141].

Early preclinical studies provided proof of concept for CD138-directed CAR-based approaches. A seminal investigation by Jiang and colleagues [125] demonstrated that NK-92MI cells (IL-2-independent derivative cell line of NK-92) engineered to express a CD138-specific CAR effectively recognized and killed CD138^+^ MM cell lines (RPMI8226, U266 and NCI-H929). The construct incorporated a CD138-binding scFv fused to a CD8α-based hinge and a CD3ζ cytoplasmic tail, which was sufficient to trigger NK activation and cytotoxic degranulation. In xenograft mouse models, CD138 CAR-NK-92MI cells significantly inhibited tumor progression and prolonged survival compared with control NK-92MI cells, without inducing overt toxicity in non-hematopoietic tissues. These data established CD138 as one of the earliest validated targets for CAR-NK therapy in MM.

More recently, an updated study by Jo et al. in Frontiers in Immunology [142] further explored the potential of CD138 CAR-NK therapy, evaluating PB-NK cells. The study confirmed potent and selective cytotoxicity against CD138^+^ myeloma cells (MM1.R and MM1.S) and demonstrated enhanced production of IFN-γ. Moreover, treatment with the histone deacetylase inhibitor entinostat (ENT) was shown to significantly enhance and sustain CAR expression in NK cells. ENT-treated CAR-NK cells demonstrated prolonged in vivo persistence and achieved greater tumor reduction in a MM xenograft model, highlighting the potential of epigenetic modulation to improve the efficacy and durability of CAR-NK cell therapy.

Together, these findings indicate that CD138 is a promising target for CAR-NK therapy, supported by its consistent overexpression in MM and favorable safety profile. The evolution of CAR constructs from NK-92 to primary NK systems may further improve persistence, in vivo expansion, and cytotoxic potency. While no clinical trials have yet been reported for CD138 CAR-NK cells, the robust preclinical data underscore their translational potential as part of the next generation of allogeneic, “off-the-shelf” immunotherapies for relapsed or refractory MM.

### 6.8. SLAMF7

SLAMF7 (also known as CS1) is a surface glycoprotein highly expressed on normal and malignant plasma cells [143] and is the target of the clinically approved monoclonal antibody elotuzumab; its consistent presence on myeloma cells and limited expression on most non-hematopoietic tissues make SLAMF7 an attractive antigen for cell-based therapies.

Early preclinical work demonstrated that a viral construct encoding a second-generation CS1-specific CAR, comprising CD28-CD3ζ intracellular signaling domains, can be expressed in human NK cells to produce CAR-NK effectors with markedly enhanced anti-myeloma activity. CS1-CAR NK cells exhibited increased cytotoxicity and IFN-γ production against MM cell lines and primary patient samples in vitro, and significantly reduced tumor burden in xenograft models relative to unmodified NK cells [124].

Reviews and subsequent analyses place these findings in context, noting that SLAMF7-directed approaches benefit from a well-characterized safety profile in humans (from antibody therapy) and are biologically rational for combination strategies that harness innate and adaptive immunity [40]. Taken together, the available evidence supports SLAMF7 as a viable target for CAR-NK development in MM, with robust preclinical activity and a translational rationale anchored by existing clinical experience with SLAMF7-directed antibodies. However, a major limitation of this strategy lies in the fact that CS1 is also expressed on normal lymphocytes and NK cells, which may lead to fratricide both in vitro and in vivo, thereby reducing NK cell viability and compromising their anti-myeloma activity.

## 7. Translation Barriers

To facilitate clinical translation of CAR-NK approaches for MM, several key translational barriers remain.

First, manufacturing scalability and product consistency are major challenges: donor-to-donor variability [12], low and variable expansion yields of primary NK cells [144], and lack of fully automated, closed-system processes [145] complicate large-scale GMP production and batch reproducibility.

Second, logistics and supply-chain constraints—including cryopreservation effects on potency, cold-chain distribution, and the need for rapid centralized or decentralized manufacturing networks—add operational complexity and cost that may limit broad access.

Third, regulatory and quality-control hurdles are nontrivial: standardizing robust potency assays, ensuring consistency among different manufacturing batches and gene-edited NK cell lines, and addressing safety aspects associated with multiplex genome editing or immortalized platforms require early and continuous interaction with regulatory authorities.

Beyond manufacturing challenges, the evolving regulatory framework for NK-cell therapies constitutes a further critical hurdle. In both the United States and Europe, manufacturers must comply with rigorous guidelines for cell-product identity, manufacturing control, and batch reproducibility. For example, in the U.S., therapies derived from human cells or tissues fall under regulations such as 21 CFR Part 1271 [146], and FDA requires validated identity assays and reproducible manufacturing across sites as a prerequisite for market authorization [147]. In Europe, the European Medicines Agency (EMA) via its Committee for Advanced Therapies (CAT) guideline emphasizes a risk-based approach with rigorous traceability, standardized testing, and critical-quality-attribute monitoring for cell-based medicinal products [148]. International harmonization efforts—for example, through the International Council for Harmonisation of Technical Requirements for Pharmaceuticals for Human Use (ICH) and the International Society for Cell & Gene Therapy (ISCT)—remain nascent, and the absence of unified global standards further increases cost, complexity, and time to market. Thus, regulatory-manufacturing interplay remains a significant translational barrier to scalable, “off-the-shelf” CAR-NK therapies.

Finally, while scalable platforms such as iPSC-derived [149,150] and well-characterized cell banks offer a promising route to “off-the-shelf” CAR-NK products, they also introduce additional manufacturing and regulatory demands—including master-cell-bank characterization [148], long-term genomic stability [151], and demonstrable control of differentiation [109,152]—which must be addressed to realize cost-effective, widely available therapies.

Collectively, these bottlenecks argue for prioritized investment in process automation, harmonized potency and release criteria, cryopreservation-compatible formulations, and early regulatory dialogue to enable the safe and scalable clinical deployment of CAR-NK therapies in MM. A concise summary of the principal translational bottlenecks is presented in Table 2.

## 8. Conclusions

Over the past two decades, the concept of enhancing NK cell functionality through CAR engineering has gained remarkable traction. A broad range of preclinical studies have explored diverse NK cell sources, culture conditions, and vector platforms, leading to the emergence of several CAR-NK candidates currently being evaluated in early-phase clinical trials for MM. These developments have firmly established CAR-NK therapy as a promising and distinct modality within the landscape of cellular immunotherapy.

However, most CAR constructs used to date were initially designed for T cells and only partially exploit NK-cell-specific signaling pathways. Given that NK cells rely on unique activating receptors and adaptor molecules to regulate their cytotoxic responses, rational CAR redesign—incorporating NK-tailored extracellular, transmembrane, and intracellular domains—may substantially enhance therapeutic potency and disease specificity. This optimization is particularly relevant in MM, where tumor heterogeneity and antigen escape pose major challenges to durable disease control.

Preclinical and early clinical evidence demonstrates that CAR-NK cells targeting MM-associated antigens such as BCMA, GPRC5D, NKG2D, CD38, CD70, CD138, and SLAMF7 can mediate potent, selective cytotoxicity with minimal toxicity. The development of iPSC-derived and multiplex-edited NK platforms has also mitigated key limitations related to persistence, cytokine dependence, and manufacturing scalability. Early clinical data show encouraging safety and biological activity without severe cytokine release or neurotoxicity, distinguishing these therapies from CAR-T cell approaches.

Nevertheless, important challenges remain, including limited in vivo expansion, potential antigen loss, and the need for sustained efficacy in heavily pretreated patients. Continued refinement of CAR design, incorporation of autocrine cytokine support, and implementation of multi-target or combination strategies are expected to enhance therapeutic performance. Collectively, current evidence supports CAR-NK cells as a safe, versatile, and scalable platform with the potential to redefine the immunotherapeutic paradigm for MM.

## Figures and Tables

**Table 1:** Current landscape of CAR-NK cell therapy trials in multiple myeloma: ongoing studies, unknown status trials, and distribution by cell source and antigen target.

NCT	Organization	Phase	Status of Study	Study’s Official Title	NK Cell Source	Target
Ongoing clinical trials						
NCT05092451	M.D. Anderson Cancer Center (Houston, TX, USA)	Phase 1/2	Recruiting	Phase I/II Study of CAR.70-Engineered IL15-transduced Cord Blood-derived NK Cells in Conjunction With Lymphodepleting Chemotherapy for the Management of Relapse/Refractory Hematological Malignances	Allogenic Cord Blood NK cells	CD70
NCT05182073	Fate Therapeutics (USA, multi-center trial)	Phase 1	Active, not recruiting	A Phase I Study of FT576 as Monotherapy and in Combination With Daratumumab in Subjects With Relapsed/Refractory Multiple Myeloma	Allogenic iPSCs	BCMA
NCT06594211	RenJi Hospital (Shanghai, Shanghai Municipality, China)	Not Applicable	Not yet recruiting	A Single-Arm, Open-Label Study of Allogeneic Anti-BCMA/GPRC5D Bispecific CAR-NK Cells (ACT-001) in Patients With Relapsed or Refractory Multiple Myeloma	Allogeneic unknown	BCMA/GPRC5D
NCT06045091	Hrain Biotechnology Co., Ltd. (Shanghai, Shanghai Municipality, China)	Early Phase 1	Recruiting	An Early Phase 1 Clinical Trial to Evaluate the Safety and Efficacy of Human BCMA Targeted CAR-NK Cells Injection for Subjects With Relapsed/Refractory Multiple Myeloma or Plasma Cell Leukemia	Unknown	BCMA
NCT06242249	Shahid Beheshti University of Medical Sciences (Tehran, Iran)	Phase 1, Phase 2	Not yet recruiting	Determining Safety and Maximum Tolerated Dose (MTD) of Anti-BCMA CAR-NK Therapy in Relapsed or Refractory Multiple Myeloma	Unknown	BCMA
NCT05498545	Second Affiliated Hospital of Xi’an Jiaotong University (Xi’an, Shaanxi, China)	Phase 1	Not yet recruiting	Universal BCMA-targeted LUCAR-B68 Cells in Patients With Relapsed/Refractory Multiple Myeloma	Unknown	BCMA
NCT06379451	Changzhou No.2 People’s Hospital (Changzhou, Jiangsu, China)	Early Phase 1	Not yet recruiting	An Exploratory Clinical Study of the Safety and Efficacy of NKG2D Chimeric Antigen Receptor NK Cell Injections for the Treatment of Refractory Recurrent Multiple Myeloma	Unknown	NKG2D
Clinical trials with unknown status						
NCT05008536	Xinqiao Hospital of Chongqing (Chongqing, Chongqing Municipality, China)	Early Phase 1	Unknown	Phase I Study to Evaluate the Safety and Effectiveness of Anti-BCMA CAR-NK Therapy in Relapsed or Refractory Multiple Myeloma	Allogenic Umbilical and Cord Blood NK Cells	BCMA
NCT03940833	Asclepius Technology Company Group (Suzhou) Co., Ltd. (Wuxi, Jiangsu, China)	Phase 1, Phase 2	Unknown	Clinical Research of Adoptive BCMA CAR-NK Cells on Relapse/Refractory MM	NK-92	BCMA
NCT05652530	Shenzhen Pregene Biopharma Co., Ltd. (Zhenghou, Henan, China)	Early Phase 1	Unknown	Clinical Study of the Safety and Efficacy of Chimeric Antigen Receptor NK Cell Injection Targeting BCMA (BCMA CAR-NK) in Patients With Relapsed/Refractory Multiple Myeloma	Unknown	BCMA

Note: Clinical trials were categorized as “ongoing” or “unknown status” according to their latest publicly available information in official register (ClinicalTrials.gov) retrieved in October 2025, to reflect the most up-to-date status of CAR-NK-cell-based studies in MM.

**Table 2:** Key translational barriers in the clinical development of CAR-NK cell therapies for multiple myeloma.

Category	Representative Barriers	Translational Impact
Manufacturing scalability and consistency	Donor-to-donor variability; limited expansion yields of primary NK cells; absence of fully automated, closed-system processes	Batch-to-batch inconsistency and limited large-scale GMP production capacity
Logistics and supply-chain constraints	Cryopreservation effects on viability and potency; complex cold-chain distribution; need for rapid, decentralized manufacturing networks	Increased cost and reduced accessibility of therapy
Regulatory and quality-control hurdles	Lack of standardized potency and identity assays; variability across manufacturing sites; evolving oversight of multiplex gene-edited NK lines	Delayed clinical translation and higher Chemistry, Manufacturing, and Controls (CMC)/regulatory burden
Technological platform limitations	iPSC-derived and immortalized NK sources require master-cell-bank characterization, genomic-stability monitoring, and differentiation control	Added safety and validation demands before “off-the-shelf” application
Global standardization gaps	Fragmented regulatory expectations between U.S., EU, and Asia; limited ICH/ISCT harmonization	Increased development cost and longer time to market

## Data Availability

No new data were created or analyzed in this study. Data sharing is not applicable to this article.

## Abbreviations

The following abbreviations are used in this manuscript:

MM	Multiple myeloma
CAR	Chimeric antigen receptor
CRS	Cytokine release syndrome
NK	Natural killer
GvHD	Graft-versus-host disease
PB	Peripheral blood
CB	Cord blood
iPSC	Induced pluripotent stem cell
BCMA	B-cell maturation antigen
ICANS	Immune effector cell–associated neurotoxicity syndrome
TAA	Tumor-associated antigens
MHC	Major histocompatibility complex
scFv	Single-chain variable fragment
TMD	Transmembrane domain
DAP10	DNAX-activating protein 10
DAP12	DNAX-activating protein 12
NCT	Number clinical trial
ADCC	Antibody-dependent cellular cytotoxicity
ESCs	Embryonic stem cells
HSPC	Hematopoietic stem and progenitor cells
ASH	American Society of Hematology
CMC	Chemistry, Manufacturing, and Controls

## References

[1] Malard F., Neri P., Bahlis N.J., Terpos E., Moukalled N., Hungria V.T.M., Manier S., Mohty M. (2024). Multiple Myeloma. Nat. Rev. Dis. Prim..

[2] Rajkumar S.V. (2024). Multiple Myeloma: 2024 Update on Diagnosis, Risk-Stratification, and Management. Am. J. Hematol..

[3] Zhuge L., Lin X., Fan Z., Jia M., Lin C., Zhu M., Teng H., Chen G. (2025). Global, Regional and National Epidemiological Trends of Multiple Myeloma from 1990 to 2021: A Systematic Analysis of the Global Burden of Disease Study 2021. Front. Public Health.

[4] Pinto V., Bergantim R., Caires H.R., Seca H., Guimarães J.E., Vasconcelos M.H. (2020). Multiple Myeloma: Available Therapies and Causes of Drug Resistance. Cancers.

[5] Vu S.H., Pham H.H., Pham T.T.P., Le T.T., Vo M.C., Jung S.H., Lee J.J., Nguyen X.H. (2023). Adoptive NK Cell Therapy-a Beacon of Hope in Multiple Myeloma Treatment. Front. Oncol..

[6] Sheykhhasan M., Ahmadieh-Yazdi A., Vicidomini R., Poondla N., Tanzadehpanah H., Dirbaziyan A., Mahaki H., Manoochehri H., Kalhor N., Dama P. (2024). CAR T Therapies in Multiple Myeloma: Unleashing the Future. Cancer Gene Ther..

[7] Gagelmann N., Riecken K., Wolschke C., Berger C., Ayuk F.A., Fehse B., Kröger N. (2020). Development of CAR-T Cell Therapies for Multiple Myeloma. Leukemia.

[8] Rendo M.J., Joseph J.J., Phan L.M., DeStefano C.B. (2022). CAR T-Cell Therapy for Patients with Multiple Myeloma: Current Evidence and Challenges. Blood Lymphat. Cancer Targets Ther..

[9] Dagar G., Gupta A., Masoodi T., Nisar S., Merhi M., Hashem S., Chauhan R., Dagar M., Mirza S., Bagga P. (2023). Harnessing the Potential of CAR-T Cell Therapy: Progress, Challenges, and Future Directions in Hematological and Solid Tumor Treatments. J. Transl. Med..

[10] Clara J.A., Childs R.W. (2022). Harnessing Natural Killer Cells for the Treatment of Multiple Myeloma. Semin. Oncol..

[11] Khawar M.B., Sun H. (2021). CAR-NK Cells: From Natural Basis to Design for Kill. Front. Immunol..

[12] Jørgensen L.V., Christensen E.B., Barnkob M.B., Barington T. (2025). The Clinical Landscape of CAR NK Cells. Exp. Hematol. Oncol..

[13] Gasteiger G., Rudensky A.Y. (2014). Interactions between Innate and Adaptive Lymphocytes. Nat. Rev. Immunol..

[14] Pishesha N., Harmand T.J., Ploegh H.L. (2022). A Guide to Antigen Processing and Presentation. Nat. Rev. Immunol..

[15] Kretschmer L., Fuchs N., Busch D.H., Buchholz V.R. (2023). Picking up Speed: Cell Cycle Regulation during Effector CD8^+^ T Cell Differentiation. Med. Microbiol. Immunol..

[16] Wang F., Cheng F., Zheng F. (2022). Stem Cell like Memory T Cells: A New Paradigm in Cancer Immunotherapy. Clin. Immunol..

[17] Mace E.M. (2023). Human Natural Killer Cells: Form, Function, and Development. J. Allergy Clin. Immunol..

[18] Chen S., Zhu H., Jounaidi Y. (2024). Comprehensive Snapshots of Natural Killer Cells Functions, Signaling, Molecular Mechanisms and Clinical Utilization. Signal Transduct. Target. Ther..

[19] Yao P., Liu Y.G., Huang G., Hao L., Wang R. (2024). The Development and Application of Chimeric Antigen Receptor Natural Killer (CAR-NK) Cells for Cancer Therapy: Current State, Challenges and Emerging Therapeutic Advances. Exp. Hematol. Oncol..

[20] Zhong Y., Liu J. (2024). Emerging Roles of CAR-NK Cell Therapies in Tumor Immunotherapy: Current Status and Future Directions. Cell Death Discov..

[21] Sterner R.C., Sterner R.M. (2021). CAR-T Cell Therapy: Current Limitations and Potential Strategies. Blood Cancer J..

[22] Peng L., Sferruzza G., Yang L., Zhou L., Chen S. (2024). CAR-T and CAR-NK as Cellular Cancer Immunotherapy for Solid Tumors. Cell. Mol. Immunol..

[23] Leivas A., Rio R., Mateos R., Paciello M.L., Garcia-Ortiz A., Fernandez L., Perez-Martinez A., Anthony Lee D., Powell D.J., Valeri A. (2018). NKG2D-CAR Transduced Primary Natural Killer Cells Efficiently Target Multiple Myeloma Cells. Blood.

[24] Ren Q., Zu Y., Su H., Lu Q., Xiang B., Luo Y., Zhang J., Song Y. (2023). Single VHH-Directed BCMA CAR-NK Cells for Multiple Myeloma. Exp. Hematol. Oncol..

[25] Park E., Mun H.J., Seo E., Hwang S., Lee J.H., Song S., Sung H., Kim H.Y., Kwon M.J. (2024). CAR NK92 Cells Targeting BCMA Can Effectively Kill Multiple Myeloma Cells Both In Vitro and In Vivo. Biomedicines.

[26] Lanier L.L. (2009). DAP10- and DAP12-Associated Receptors in Innate Immunity. Immunol. Rev..

[27] Upshaw J.L., Arneson L.N., Schoon R.A., Dick C.J., Billadeau D.D., Leibson P.J. (2006). NKG2D-Mediated Signaling Requires a DAP10-Bound Grb2-Vav1 Intermediate and Phosphatidylinositol-3-Kinase in Human Natural Killer Cells. Nat. Immunol..

[28] Wang K., Zhang Y., Han Z., Wang Y. (2025). Does a Natural Killer Need a CAR?. Front. Immunol..

[29] Wrona E., Borowiec M., Potemski P. (2021). CAR-NK Cells in the Treatment of Solid Tumors. Int. J. Mol. Sci..

[30] Li Y., Hermanson D.L., Moriarity B.S., Kaufman D.S. (2018). Human IPSC-Derived Natural Killer Cells Engineered with Chimeric Antigen Receptors Enhance Anti-Tumor Activity. Cell Stem Cell.

[31] Xu Y., Liu Q., Zhong M., Wang Z., Chen Z., Zhang Y., Xing H., Tian Z., Tang K., Liao X. (2019). 2B4 Costimulatory Domain Enhancing Cytotoxic Ability of Anti-CD5 Chimeric Antigen Receptor Engineered Natural Killer Cells against T Cell Malignancies. J. Hematol. Oncol..

[32] Van den Eynde A., Gehrcken L., Verhezen T., Lau H.W., Hermans C., Lambrechts H., Flieswasser T., Quatannens D., Roex G., Zwaenepoel K. (2024). IL-15-Secreting CAR Natural Killer Cells Directed toward the Pan-Cancer Target CD70 Eliminate Both Cancer Cells and Cancer-Associated Fibroblasts. J. Hematol. Oncol..

[33] Liu E., Marin D., Banerjee P., Macapinlac H.A., Thompson P., Basar R., Nassif Kerbauy L., Overman B., Thall P., Kaplan M. (2020). Use of CAR-Transduced Natural Killer Cells in CD19-Positive Lymphoid Tumors. N. Engl. J. Med..

[34] Page A., Chuvin N., Valladeau-Guilemond J., Depil S. (2024). Development of NK Cell-Based Cancer Immunotherapies through Receptor Engineering. Cell. Mol. Immunol..

[35] Hudecek M., Sommermeyer D., Kosasih P.L., Silva-Benedict A., Liu L., Rader C., Jensen M.C., Riddell S.R. (2015). The Nonsignaling Extracellular Spacer Domain of Chimeric Antigen Receptors Is Decisive for in Vivo Antitumor Activity. Cancer Immunol. Res..

[36] Sharma P., Kanapuru B., George B., Lin X., Xu Z., Bryan W.W., Pazdur R., Theoret M.R. (2022). FDA Approval Summary: Idecabtagene Vicleucel for Relapsed or Refractory Multiple Myeloma. Clin. Cancer Res..

[37] Natrajan K., Kaushal M., George B., Kanapuru B., Theoret M.R. (2024). FDA Approval Summary: Ciltacabtagene Autoleucel for Relapsed or Refractory Multiple Myeloma. Clin. Cancer Res..

[38] Depil S., Duchateau P., Grupp S.A., Mufti G., Poirot L. (2020). ‘Off-the-Shelf’ Allogeneic CAR T Cells: Development and Challenges. Nat. Rev. Drug Discov..

[39] Li Y.R., Zhu Y., Fang Y., Lyu Z., Yang L. (2025). Emerging Trends in Clinical Allogeneic CAR Cell Therapy. Med.

[40] Daher M., Melo Garcia L., Li Y., Rezvani K. (2021). CAR-NK Cells: The next Wave of Cellular Therapy for Cancer. Clin. Transl. Immunol..

[41] Parikh R.H., Lonial S. (2023). Chimeric Antigen Receptor T-cell Therapy in Multiple Myeloma: A Comprehensive Review of Current Data and Implications for Clinical Practice. CA Cancer J. Clin..

[42] Mansouri V., Yazdanpanah N., Rezaei N. (2022). The Immunologic Aspects of Cytokine Release Syndrome and Graft versus Host Disease Following CAR T Cell Therapy. Int. Rev. Immunol..

[43] Morris E.C., Neelapu S.S., Giavridis T., Sadelain M. (2022). Cytokine Release Syndrome and Associated Neurotoxicity in Cancer Immunotherapy. Nat. Rev. Immunol..

[44] Kikuchi T., Tsukada N., Kunisada K., Nomura-Yogo M., Ishida T. (2024). Local Cytokine Release Syndrome After Idecabtagene Vicleucel Therapy in Patients With Multiple Myeloma: Two Case Reports. Cureus.

[45] Cheng H., Shao L., Wang D., Chen Y., Sun Y., Chen Z., Liu J., Wang X., Chen W., Sang W. (2025). Comprehensive Characterization of Cytopenia after Chimeric Antigen Receptor-T Cell Infusion in Patients with Relapsed or Refractory Multiple Myeloma. Cytotherapy.

[46] Sperling A.S. (2024). Cytopenias in BCMA CAR T: Unraveling Inflammatory Mechanisms. Blood Adv..

[47] Sanber K., Savani B., Jain T. (2021). Graft-versus-Host Disease Risk after Chimeric Antigen Receptor T-Cell Therapy: The Diametric Opposition of T Cells. Br. J. Haematol..

[48] Yang R., Yang Y., Liu R., Wang Y., Yang R., He A. (2024). Advances in CAR-NK Cell Therapy for Hematological Malignancies. Front. Immunol..

[49] Zhang B., Yang M., Zhang W., Liu N., Wang D., Jing L., Xu N., Yang N., Ren T. (2024). Chimeric Antigen Receptor-Based Natural Killer Cell Immunotherapy in Cancer: From Bench to Bedside. Cell Death Dis..

[50] Harris D.T., Hager M.V., Smith S.N., Cai Q., Stone J.D., Kruger P., Lever M., Dushek O., Schmitt T.M., Greenberg P.D. (2018). Comparison of T Cell Activities Mediated by Human TCRs and CARs That Use the Same Recognition Domains. J. Immunol..

[51] Salter A.I., Rajan A., Kennedy J.J., Ivey R.G., Shelby S.A., Leung I., Templeton M.L., Muhunthan V., Voillet V., Sommermeyer D. (2022). Comparative Analysis of TCR and CAR Signaling Informs CAR Designs with Superior Antigen Sensitivity and in Vivo Function. Sci. Signal..

[52] Goodridge J.P., Bjordahl R., Mahmood S., Reiser J., Gaidarova S., Blum R., Cichocki F., Chu H., Bonello G., Lee T. (2020). FT576: Multi-Specific Off-the-Shelf CAR-NK Cell Therapy Engineered for Enhanced Persistence, Avoidance of Self-Fratricide and Optimized Mab Combination Therapy to Prevent Antigenic Escape and Elicit a Deep and Durable Response in Multiple Myeloma. Blood.

[53] Ullrich E., Salzmann-Manrique E., Bakhtiar S., Bremm M., Gerstner S., Herrmann E., Bader P., Hoffmann P., Holler E., Edinger M. (2016). Relation between Acute GVHD and NK Cell Subset Reconstitution Following Allogeneic Stem Cell Transplantation. Front. Immunol..

[54] Shimasaki N., Jain A., Campana D. (2020). NK Cells for Cancer Immunotherapy. Nat. Rev. Drug Discov..

[55] Lamb M.G., Rangarajan H.G., Tullius B.P., Lee D.A. (2021). Natural Killer Cell Therapy for Hematologic Malignancies: Successes, Challenges, and the Future. Stem Cell Res. Ther..

[56] Li Y.R., Fang Y., Niu S., Chen Y., Lyu Z., Yang L. (2025). Managing Allorejection in Off-the-Shelf CAR-Engineered Cell Therapies. Mol. Ther..

[57] Liu F., Tarannum M., Zhao Y., Zhang Y.J., Ham J.D., Lei K., Qiang Y., Deng X., Nguyen M., Dinh K. (2025). Selective HLA Knockdown and PD-L1 Expression Prevent Allogeneic CAR-NK Cell Rejection and Enhance Safety and Anti-Tumor Responses in Xenograft Mice. Nat. Commun..

[58] Hammer Q., Perica K., Mbofung R.M., van Ooijen H., Martin K.E., Momayyezi P., Varady E., Pan Y., Jelcic M., Groff B. (2024). Genetic Ablation of Adhesion Ligands Mitigates Rejection of Allogeneic Cellular Immunotherapies. Cell Stem Cell.

[59] Kim H. (2025). Overcoming Immune Barriers in Allogeneic CAR-NK Therapy: From Multiplex Gene Editing to AI-Driven Precision Design. Biomolecules.

[60] Ghobadi A., Bachanova V., Patel K., Park J.H., Flinn I., Riedell P.A., Bachier C., Diefenbach S.C., Wong C., Bickers C. (2025). Induced Pluripotent Stem-Cell-Derived CD19-Directed Chimeric Antigen Receptor Natural Killer Cells in B-Cell Lymphoma: A Phase 1, First-in-Human Trial. Lancet.

[61] Dhakal B., Berdeja J.G., Gregory T., Ly T., Bickers C., Zong X., Wong L., Goodridge J.P., Cooley S., Valamehr B. (2022). Interim Phase I Clinical Data of FT576 As Monotherapy and in Combination with Daratumumab in Subjects with Relapsed/Refractory Multiple Myeloma. Blood.

[62] Marofi F., Saleh M.M., Rahman H.S., Suksatan W., Al-Gazally M.E., Abdelbasset W.K., Thangavelu L., Yumashev A.V., Hassanzadeh A., Yazdanifar M. (2021). CAR-Engineered NK Cells; a Promising Therapeutic Option for Treatment of Hematological Malignancies. Stem Cell Res. Ther..

[63] O’Neal J., Cooper M.L., Ritchey J.K., Gladney S., Niswonger J., González L.S., Street E., Haas G.J., Carter A., Amayta P.N. (2023). Anti-Myeloma Efficacy of CAR-INKT Is Enhanced with a Long-Acting IL-7, RhIL-7-HyFc. Blood Adv..

[64] Fiorenza S., Ritchie D.S., Ramsey S.D., Turtle C.J., Roth J.A. (2020). Value and Affordability of CAR T-Cell Therapy in the United States. Bone Marrow Transplant..

[65] Jagannath S., Joseph N., Crivera C., Kharat A., Jackson C.C., Valluri S., Cost P., Phelps H., Slowik R., Klein T. (2023). Component Costs of CAR-T Therapy in Addition to Treatment Acquisition Costs in Patients with Multiple Myeloma. Oncol. Ther..

[66] Nahi H., Chrobok M., Meinke S., Gran C., Marquardt N., Afram G., Sutlu T., Gilljam M., Stellan B., Wagner A.K. (2022). Autologous NK Cells as Consolidation Therapy Following Stem Cell Transplantation in Multiple Myeloma. Cell Rep. Med..

[67] D’Souza C., Keam S.P., Yeang H.X.A., Neeson M., Richardson K., Hsu A.K., Canfield R., Bezman N., Robbins M., Quach H. (2021). Myeloma Natural Killer Cells Are Exhausted and Have Impaired Regulation of Activation. Haematologica.

[68] Pazina T., MacFarlane A.W., Bernabei L., Dulaimi E., Kotcher R., Yam C., Bezman N.A., Robbins M.D., Ross E.A., Campbell K.S. (2021). Alterations of Nk Cell Phenotype in the Disease Course of Multiple Myeloma. Cancers.

[69] Tahri S., de Jong M.M.E., Fokkema C., Papazian N., Kellermayer Z., Vermeulen M., van Duin M., van de Woestijne P., Nasserinejad K., Saraci E. (2022). Single-Cell Transcriptomic Analysis Reveals Reduction of Cytotoxic NK Cells in a Subset of Newly Diagnosed Multiple Myeloma Patients Impacting Outcome after Daratumumab Therapy. Blood.

[70] Wu S.Y., Fu T., Jiang Y.Z., Shao Z.M. (2020). Natural Killer Cells in Cancer Biology and Therapy. Mol. Cancer.

[71] Lamers-Kok N., Panella D., Georgoudaki A.M., Liu H., Özkazanc D., Kučerová L., Duru A.D., Spanholtz J., Raimo M. (2022). Natural Killer Cells in Clinical Development as Non-engineered, Engineered, and Combination Therapies. J. Hematol. Oncol..

[72] Berrien-Elliott M.M., Jacobs M.T., Fehniger T.A. (2023). Allogeneic Natural Killer Cell Therapy. Blood.

[73] Kennedy P.R., Felices M., Miller J.S. (2022). Challenges to the Broad Application of Allogeneic Natural Killer Cell Immunotherapy of Cancer. Stem Cell Res. Ther..

[74] Liu M., Meng Y., Zhang L., Han Z., Feng X. (2021). High-Efficient Generation of Natural Killer Cells from Peripheral Blood with Preferable Cell Vitality and Enhanced Cytotoxicity by Combination of IL-2, IL-15 and IL-18. Biochem. Biophys. Res. Commun..

[75] Boje A.S., Langner A., Gehlert C.L., Reitinger C., Nimmerjahn F., Murga Penas E.M., Bendig S., Chitadze G., Brüggemann M., Diemer K. (2024). A Novel Platform Technology for the Development of NK Cell-Based Cellular Immunotherapies. Blood.

[76] Reina-Ortiz C., Constantinides M., Fayd-Herbe-de-Maudave A., Présumey J., Hernandez J., Cartron G., Giraldos D., Díez R., Izquierdo I., Azaceta G. (2021). Expanded NK Cells from Umbilical Cord Blood and Adult Peripheral Blood Combined with Daratumumab Are Effective against Tumor Cells from Multiple Myeloma Patients. Oncoimmunology.

[77] Woll P.S., Grzywacz B., Tian X., Marcus R.K., Knorr D.A., Verneris M.R., Kaufman D.S. (2009). Human Embryonic Stem Cells Differentiate into a Homogeneous Population of Natural Killer Cells with Potent in Vivo Antitumor Activity. Blood.

[78] Knorr D.A., Ni Z., Hermanson D., Hexum M.K., Bendzick L., Cooper L.J.N., Lee D.A., Kaufman D.S. (2013). Clinical-Scale Derivation of Natural Killer Cells From Human Pluripotent Stem Cells for Cancer Therapy. Stem Cells Transl. Med..

[79] Zhu H., Kaufman D.S. (2019). An Improved Method to Produce Clinical-Scale Natural Killer Cells from Human Pluripotent Stem Cells. In Vitro Differentiation of T-Cells: Methods and Protocols.

[80] Luevano M., Madrigal A., Saudemont A. (2012). Generation of Natural Killer Cells from Hematopoietic Stem Cells in Vitro for Immunotherapy. Cell. Mol. Immunol..

[81] Fabian K.P., Hodge J.W. (2021). The Emerging Role of Off-the-Shelf Engineered Natural Killer Cells in Targeted Cancer Immunotherapy. Mol. Ther. Oncolytics.

[82] Shereck E., Day N.S., Awasthi A., Ayello J., Chu Y., McGuinn C., van de Ven C., Lim M.S., Cairo M.S. (2019). Immunophenotypic, Cytotoxic, Proteomic and Genomic Characterization of Human Cord Blood vs. Peripheral Blood CD56^Dim^ NK Cells. Innate Immun..

[83] Kroll K., Reeves R.K. (2023). Protocol for Identification and Computational Analysis of Human Natural Killer Cells Using Flow Cytometry and R. STAR Protoc..

[84] Angelo L.S., Banerjee P.P., Monaco-Shawver L., Rosen J.B., Makedonas G., Forbes L.R., Mace E.M., Orange J.S. (2015). Practical NK Cell Phenotyping and Variability in Healthy Adults. Immunol. Res..

[85] Porrata L.F. (2022). Natural Killer Cells Are Key Host Immune Effector Cells Affecting Survival in Autologous Peripheral Blood Hematopoietic Stem Cell Transplantation. Cells.

[86] Dogra P., Rancan C., Ma W., Toth M., Senda T., Carpenter D.J., Kubota M., Matsumoto R., Thapa P., Szabo P.A. (2020). Tissue Determinants of Human NK Cell Development, Function, and Residence. Cell.

[87] Zhang Y., Wallace D.L., De Lara C.M., Ghattas H., Asquith B., Worth A., Griffin G.E., Taylor G.P., Tough D.F., Beverley P.C.L. (2007). In Vivo Kinetics of Human Natural Killer Cells: The Effects of Ageing and Acute and Chronic Viral Infection. Immunology.

[88] Caligiuri M.A. (2008). Human Natural Killer Cells. Blood.

[89] Imai C., Iwamoto S., Campana D. (2005). Genetic Modification of Primary Natural Killer Cells Overcomes Inhibitory Signals and Induces Specific Killing of Leukemic Cells. Blood.

[90] Ng Y.Y., Du Z., Zhang X., Chng W.J., Wang S. (2022). CXCR4 and Anti-BCMA CAR Co-Modified Natural Killer Cells Suppress Multiple Myeloma Progression in a Xenograft Mouse Model. Cancer Gene Ther..

[91] Cao Z., Yang C., Wang Y., Wang C., Wang Q., Ye G., Liu T., Wang Q., Wang H., Gong Y. (2022). Allogeneic CAR-NK Cell Therapy Targeting Both BCMA and GPRC5D for the Treatment of Multiple Myeloma. Blood.

[92] Huang R., Wen Q., Zhang X. (2023). CAR-NK Cell Therapy for Hematological Malignancies: Recent Updates from ASH 2022. J. Hematol. Oncol..

[93] Kotylo P.K., Baenzinger J.C., Yoder M.C., Engle W.A., Bolinger C.D. (1990). Rapid Analysis of Lymphocyte Subsets in Cord Blood. Am. J. Clin. Pathol..

[94] Li L., Chen H., Marin D., Xi Y., Miao Q., Lv J., Banerjee P.P., Shaim H., Daher M., Basar R. (2019). A Novel Immature Natural Killer Cell Subpopulation Predicts Relapse after Cord Blood Transplantation. Blood Adv..

[95] Luevano M., Daryouzeh M., Alnabhan R., Querol S., Khakoo S., Madrigal A., Saudemont A. (2012). The Unique Profile of Cord Blood Natural Killer Cells Balances Incomplete Maturation and Effective Killing Function upon Activation. Hum. Immunol..

[96] Alnabhan R., Madrigal A., Saudemont A. (2015). Differential Activation of Cord Blood and Peripheral Blood Natural Killer Cells by Cytokines. Cytotherapy.

[97] Liu E., Ang S.O.T., Kerbauy L., Basar R., Kaur I., Kaplan M., Li L., Tong Y., Daher M., Ensley E.L. (2021). GMP-Compliant Universal Antigen Presenting Cells (UAPC) Promote the Metabolic Fitness and Antitumor Activity of Armored Cord Blood CAR-NK Cells. Front. Immunol..

[98] Castellano E., García-Ortiz A., Ugalde L., Maroto Martin E., Encinas Mayoral J., Oliva R., Garcia-Garcia L., Peña I., Álvarez N., Carreño G. (2024). CRISPR/Cas9 Multi-Editing Enhances CAR NK Cells Therapeutic Potential Against Multiple Myeloma. Blood.

[99] Lin P., Reyes Silva F.C., Lin P., Gilbert A.L., Acharya S., Nunez Cortes A.K., Banerjee P., Fang D., Melo Garcia L., Daher M. (2023). CD70 CAR NK Cells in the Treatment of Multiple Myeloma. Blood.

[100] Veluchamy J. (2020). An off the Shelf, GMP Compliant, Fully Closed and Semi-Automated Large-Scale Production System for Allogeneic NK Cells. Cytotherapy.

[101] Spanholtz J., Preijers F., Tordoir M., Trilsbeek C., Paardekooper J., de Witte T., Schaap N., Dolstra H. (2011). Clinical-Grade Generation of Active NK Cells from Cord Blood Hematopoietic Progenitor Cells for Immunotherapy Using a Closed-System Culture Process. PLoS ONE.

[102] Somanchi S., Guo X., He S., Mathur R., Difiglia A., Rana H., Ling W., Edinger J., Kaufmann G.F., Zeldis J.B. (2019). Development of CD38 CAR Engineered Human Placental Hematopoietic Stem Cell Derived Natural Killer Cells (PNK-CAR38) As Allogeneic Cancer Immunotherapy. Blood.

[103] Goldenson B.H., Zhu H., Wang Y.Z.M., Heragu N., Bernareggi D., Ruiz-Cisneros A., Bahena A., Ask E.H., Hoel H.J., Malmberg K.J. (2020). Umbilical Cord Blood and IPSC-Derived Natural Killer Cells Demonstrate Key Differences in Cytotoxic Activity and KIR Profiles. Front. Immunol..

[104] van Hauten P.M.M., Hooijmaijers L., Vidal-Manrique M., van der Waart A.B., Hobo W., Wu J., Blijlevens N.M.A., Jansen J.H., Walcheck B., Schaap N.P.M. (2024). Engineering of CD34^+^ Progenitor-Derived Natural Killer Cells with Higher-Affinity CD16a for Enhanced Antibody-Dependent Cellular Cytotoxicity. Cytotherapy.

[105] Holstein S.A., Cooley S., Hari P., Jagannath S., Balint C.R., van Der Touw W., Donato M.L., McCarthy P.L., Wallace P.K., Zhang X. (2019). Results of a Phase I Study of Pnk-007, Allogeneic, Off the Shelf NK Cell, Post Autologous Transplant in Multiple Myeloma (NCT02955550). Blood.

[106] van Der Touw W., Kang L., Tario J.D., Stout B., Voskinarian-Berse V., Rousseva V., Wallace P.K., Hariri R., Zhang X.P. (2019). Immune Monitoring of CD34^+^ Placental Cell Derived Natural Killer Cell Therapy (PNK-007) in Phase I Study of Multiple Myeloma. Blood.

[107] Ilic D., Ogilvie C. (2022). Pluripotent Stem Cells in Clinical Setting-New Developments and Overview of Current Status. Stem Cells.

[108] Karagiannis P., Kim S.I. (2021). IPSC-Derived Natural Killer Cells for Cancer Immunotherapy. Mol. Cells.

[109] Cichocki F., van der Stegen S.J.C., Miller J.S. (2023). Engineered and Banked IPSCs for Advanced NK- and T-Cell Immunotherapies. Blood.

[110] Bock A.M., Knorr D., Kaufman D.S. (2013). Development, Expansion, and In Vivo Monitoring of Human NK Cells from Human Embryonic Stem Cells (HESCs) and and Induced Pluripotent Stem Cells (IPSCs). J. Vis. Exp..

[111] Euchner J., Sprissler J., Cathomen T., Fürst D., Schrezenmeier H., Debatin K.M., Schwarz K., Felgentreff K. (2021). Natural Killer Cells Generated From Human Induced Pluripotent Stem Cells Mature to CD56brightCD16^+^NKp80^+/−^ In-Vitro and Express KIR2DL2/DL3 and KIR3DL1. Front. Immunol..

[112] Poetsch M.S., Strano A., Guan K. (2022). Human Induced Pluripotent Stem Cells: From Cell Origin, Genomic Stability, and Epigenetic Memory to Translational Medicine. Stem Cells.

[113] Kim K., Doi A., Wen B., Ng K., Zhao R., Cahan P., Kim J., Aryee M., Ji H., Ehrlich L. (2010). Epigenetic Memory in Induced Pluripotent Stem Cells. Nature.

[114] Woan K.V., Kim H., Bjordahl R., Davis Z.B., Gaidarova S., Goulding J., Hancock B., Mahmood S., Abujarour R., Wang H. (2021). Harnessing Features of Adaptive NK Cells to Generate IPSC-Derived NK Cells for Enhanced Immunotherapy. Cell Stem Cell.

[115] Goodridge J.P., Bjordahl R., Mahmood S., Reiser J., Gaidarova S., Blum R., Cichocki F., Chu H., Bonello G., Lee T. (2021). FT576 Path to First-of-Kind Clinical Trial: Translation of a Versatile Multi-Antigen Specific off-the-Shelf NK Cell for Treatment of Multiple Myeloma. Cancer Res..

[116] Cichocki F., Bjordahl R., Goodridge J.P., Mahmood S., Gaidarova S., Abujarour R., Davis Z.B., Merino A., Tuininga K., Wang H. (2022). Quadruple Gene-Engineered Natural Killer Cells Enable Multi-Antigen Targeting for Durable Antitumor Activity against Multiple Myeloma. Nat. Commun..

[117] ATCC NK-92^®^. https://www.atcc.org/products/crl-2407.

[118] Gunesch J.Y., Angelo L.S., Mahapatra S., Deering R.P., Kowalko J.E., Sleiman P., Tobias J.W., Monaco-Shawver L., Orange J.S., Mace E.M. (2019). Genome-Wide Analyses and Functional Profiling of Human NK Cell Lines. Mol. Immunol..

[119] Klingemann H. (2025). The Natural Killer Cell Line NK-92 and Its Genetic Variants: Impact on NK Cell Research and Cancer Immunotherapy. Cancers.

[120] Klingemann H. (2023). The NK-92 Cell Line—30 Years Later: Its Impact on Natural Killer Cell Research and Treatment of Cancer. Cytotherapy.

[121] Snyder K.M., Hullsiek R., Mishra H.K., Mendez D.C., Li Y., Rogich A., Kaufman D.S., Wu J., Walcheck B. (2018). Expression of a Recombinant High Affinity IgG Fc Receptor by Engineered NK Cells as a Docking Platform for Therapeutic MAbs to Target Cancer Cells. Front. Immunol..

[122] Huang R.S., Shih H.A., Lai M.C., Chang Y.J., Lin S. (2020). Enhanced NK-92 Cytotoxicity by CRISPR Genome Engineering Using Cas9 Ribonucleoproteins. Front. Immunol..

[123] Navarrete-Galvan L., Guglielmo M., Cruz Amaya J., Smith-Gagen J., Lombardi V.C., Merica R., Hudig D. (2022). Optimizing NK-92 Serial Killers: Gamma Irradiation, CD95/Fas-Ligation, and NK or LAK Attack Limit Cytotoxic Efficacy. J. Transl. Med..

[124] Chu J., Deng Y., Benson D.M., He S., Hughes T., Zhang J., Peng Y., Mao H., Yi L., Ghoshal K. (2014). CS1-Specific Chimeric Antigen Receptor (CAR)-Engineered Natural Killer Cells Enhance in Vitro and in Vivo Antitumor Activity against Human Multiple Myeloma. Leukemia.

[125] Jiang H., Zhana W., Shang P., Zhana H., Fu W., Ye F., Zeng T., Huanga H., Zhang X., Sun W. (2014). Transfection of Chimeric Anti-CD138 Gene Enhances Natural Killer Cell Activation and Killing of Multiple Myeloma Cells. Mol. Oncol..

[126] Maroto-Martín E., Encinas J., García-Ortiz A., Alonso R., Leivas A., Paciello M.L., Garrido V., Cedena T., Ugalde L., Powell D.J. (2019). PS1209 NKG2D and BCMA-CAR NK cells efficiently eliminate multiple myeloma cells. A comprehensive comparison between two clinically relevant CARs. HemaSphere.

[127] Laurent S.A., Hoffmann F.S., Kuhn P.-H., Cheng Q., Chu Y., Schmidt-Supprian M., Hauck S.M., Schuh E., Krumbholz M., Rubsamen H. (2015). G-Secretase Directly Sheds the Survival Receptor BCMA from Plasma Cells. Nat. Commun..

[128] Motais B., Charvátová S., Walek Z., Hájek R., Bagó J.R. (2023). NK92 Expressing Anti-BCMA CAR and Secreted TRAIL for the Treatment of Multiple Myeloma: Preliminary In Vitro Assessment. Cells.

[129] Fate Therapeutics Fate Therapeutics Reports First Quarter 2024 Financial Results and Business Updates. https://ir.fatetherapeutics.com/news-releases/news-release-details/fate-therapeutics-reports-first-quarter-2024-financial-results.

[130] Mikkilineni L., Kochenderfer J.N. (2017). Chimeric Antigen Receptor T-Cell Therapies for Multiple Myeloma. Blood.

[131] Roex G., Campillo-Davo D., Flumens D., Shaw P.A.G., Krekelbergh L., De Reu H., Berneman Z.N., Lion E., Anguille S. (2022). Two for One: Targeting BCMA and CD19 in B-Cell Malignancies with off-the-Shelf Dual-CAR NK-92 Cells. J. Transl. Med..

[132] Zhou D., Wang Y., Chen C., Li Z., Xu K., Zhao K. (2024). Targeting GPRC5D for Multiple Myeloma Therapy. J. Hematol. Oncol..

[133] Yang C., Wang Y., Liu T., Wang C., Wang H., Wang Q., Wang Q., Ye G., Tang R., Cao Z. (2023). Dual-Targeted CAR-NK Cell Therapy: Optimized CAR Design to Prevent Antigen Escape and Elicit a Deep and Durable Response in Multiple Myeloma. Cancer Res..

[134] Fu J., Jiang L., Zhu Z., Yan Y., Wu G., Wei M., Ning J., Yang J. (2023). Efficacy of Human IPSC-Derived CAR-NK Cells Targeting Multiple Myeloma Cells. Blood.

[135] Leivas A., Valeri A., Córdoba L., García-Ortiz A., Ortiz A., Sánchez-Vega L., Graña-Castro O., Fernández L., Carreño-Tarragona G., Pérez M. (2021). NKG2D-CAR-Transduced Natural Killer Cells Efficiently Target Multiple Myeloma. Blood Cancer J..

[136] Gozzetti A., Ciofini S., Simoncelli M., Santoni A., Pacelli P., Raspadori D., Bocchia M. (2022). Anti CD38 Monoclonal Antibodies for Multiple Myeloma Treatment. Hum. Vaccines Immunother..

[137] de Acha O.P., Reiman L., Jayabalan D.S., Walker Z.J., Bosma G., Keller A.L., Parzych S.E., Abbott D., Idler B.M., Ribadeneyra D. (2023). CD38 Antibody Re-Treatment in Daratumumab-Refractory Multiple Myeloma after Time on Other Therapies. Blood Adv..

[138] Karvouni M., Vidal-Manrique M., Susek K.H., Hussain A., Gilljam M., Zhang Y., Gray J.D., Lund J., Kaufmann G., Ljunggren H.-G. (2023). Challenges in αCD38-Chimeric Antigen Receptor (CAR)-Expressing Natural Killer (NK) Cell-Based Immunotherapy in Multiple Myeloma: Harnessing the CD38dim Phenotype of Cytokine-Stimulated NK Cells as a Strategy to Prevent Fratricide. Cytotherapy.

[139] Reiser J., Ruby Chan S., Mathavan K., Sillitti D., Mottershead C., Mattson B., Pache M., Gutierrez A., Scoon W., Zhu Y. (2022). FT555: Off-the-Shelf CAR-NK Cell Therapy Co-Targeting GPRC5D and CD38 for the Treatment of Multiple Myeloma. Blood.

[140] Akhmetzyanova I., McCarron M.J., Parekh S., Chesi M., Bergsagel P.L., Fooksman D.R. (2020). Dynamic CD138 Surface Expression Regulates Switch between Myeloma Growth and Dissemination. Leukemia.

[141] Li H., Song W., Li Z., Zhang M. (2022). Preclinical and Clinical Studies of CAR-NK-Cell Therapies for Malignancies. Front. Immunol..

[142] Jo D.H., Kaczmarek S., Khan A.U.H., Pervin J., Clark D.M., Gadde S., Wang L., McComb S., Visram A., Lee S.H. (2025). Entinostat, a Histone Deacetylase Inhibitor, Enhances CAR-NK Cell Anti-Tumor Activity by Sustaining CAR Expression. Front. Immunol..

[143] Hsi E.D., Steinle R., Balasa B., Szmania S., Draksharapu A., Shum B.P., Huseni M., Powers D., Nanisetti A., Zhang Y. (2008). CS1, a Potential New Therapeutic Antibody Target for the Treatment of Multiple Myeloma. Clin. Cancer Res..

[144] Pfefferle A., Contet J., Wong K., Chen C., Verhoeyen E., Slichter C.K., Schluns K.S., Cursons J., Berry R., Nikolic I. (2024). Optimisation of a Primary Human CAR-NK Cell Manufacturing Pipeline. Clin. Transl. Immunol..

[145] Kikuchi T., Takeuchi I., Yamaguchi H. (2025). Scalable Production Process Development for NK Cells Targeting Large-Scale Expansion. Regen. Ther..

[146] Food and Drug Administration. https://www.govinfo.gov/content/pkg/CFR-2011-title21-vol8/pdf/CFR-2011-title21-vol8-part1271.pdf.

[147] Lowdell M.W. (2025). Considerations for Manufacturing of Cell and Gene Medicines for Clinical Development. Cytotherapy.

[148] Europesn Medicines Agency Guideline on Quality, Non-Clinical and Clinical Requirements for Investigational Advanced Therapy Medicinal Products in Clinical Trials-Scientific Guideline. https://www.ema.europa.eu/en/guideline-quality-non-clinical-clinical-requirements-investigational-advanced-therapy-medicinal-products-clinical-trials-scientific-guideline.

[149] Lu S.-J., Feng F. (2021). CAR-NK Cells from Engineered Pluripotent Stem Cells: Off-the-Shelf Therapeutics for All Patients. Stem Cells Transl. Med..

[150] Dhaiban S., Chandran S., Noshi M., Sajini A.A. (2025). Clinical Translation of Human IPSC Technologies: Advances, Safety Concerns, and Future Directions. Front. Cell Dev. Biol..

[151] Madrid M., Lakshmipathy U., Zhang X., Bharti K., Wall D.M., Sato Y., Muschler G., Ting A., Smith N., Deguchi S. (2024). Considerations for the Development of iPSC-Derived Cell Therapies: A Review of Key Challenges by the JSRM-ISCT iPSC Committee. Cytotherapy.

[152] Kwon D., Moon B.K., Han M., Lee T., Lee J., Kang K. (2024). Genetically Stable Multi-Gene Edited iPSCs-Derived NK Cells for Enhanced Cancer Immunotherapy. Mol. Ther. Oncolytics.

